# Targeting TYK2 alleviates Rab27A-induced malignant progression of non-small cell lung cancer via disrupting IFNα-TYK2-STAT-HSPA5 axis

**DOI:** 10.1038/s41698-024-00574-1

**Published:** 2024-03-23

**Authors:** Yuanyuan Zeng, Jian Zhao, Zhengyan Wu, Yongkang Huang, Anqi Wang, Jianjie Zhu, Mengmeng Xu, Weijie Zhang, Xiaohui Zhang, Jianjun Li, Jian-an Huang, Zeyi Liu

**Affiliations:** 1 Department of Pulmonary and Critical Care Medicine, The First Affiliated Hospital of Soochow University, 215006 Suzhou, China; 2 Institute of Respiratory Diseases, Soochow University, 215006 Suzhou, China; 3 Suzhou Key Laboratory for Respiratory Diseases, 215006 Suzhou, China; 4 Department of Health Management Center, The First Affiliated Hospital of Soochow University, Suzhou, 215006 Suzhou, China; 5 Department of Pathology, The Second Affiliated Hospital of Soochow University, 215006 Suzhou, China

**Keywords:** Targeted therapies, Non-small-cell lung cancer

## Abstract

Rab27A is a small GTPase-mediating exosome secretion, which participates in tumorigenesis of multiple cancer types. Understanding the biological role of Rab27A in non-small cell lung cancer (NSCLC) is of great importance for oncological research and clinical treatment. In this study, we investigate the function and internal mechanism of Rab27A in NSCLC. Results show that Rab27A is overexpressed in NSCLC, and regulates the tumor proliferation, migration, invasion, and cell motility in vitro and in vivo, and is negatively regulated by miR-124. Further research reveals that upregulated Rab27A can induce the production of IFNα in the medium by mediating exosome secretion. Then IFNα activates TYK2/STAT/HSPA5 signaling to promote NSCLC cell proliferation and metastasis. This process can be suppressed by TYK2 inhibitor Cerdulatinib. These results suggest that Rab27A is involved in the pathogenesis of NSCLC by regulating exosome secretion and downstream signaling, and inhibitors targeting this axis may become a promising strategy in future clinical practice.

## Introduction

The early screening and treatment of non-small cell lung cancer (NSCLC) is a global challenge^1^. Recently, multiple treatment methods have been widely used in clinical practice^1^. Nevertheless, difficulties like drug resistance are unavoidable^2^. Therefore, elucidation of the mechanisms underlying NSCLC tumorigenesis is of great importance and urgency.

The *RAB* genes were discovered in the 1980s, and subsequent studies have shown that Rabs participate in intracellular transportation, autophagy, and transmembrane signal transduction in eukaryotes^3–5^. Several studies have suggested that *RAB* genes are involved in the tumorigenesis of several malignancies^6–8^. Rab27A is a Rab family member that is mainly responsible for the transmembrane transport of cellular substances, mediating vesicle endocytosis and exocytosis^9,10^. Previous studies have shown that *RAB27A* promotes the malignant biological behavior of tumor cells by enhancing the secretion of chemokines, metalloproteases, and exosomes^9^.

Tyrosine kinase 2 (*TYK2*) is an intermediator that participates in cytokine signaling and downstream STAT (Signal transducer and activator of transcription)-induced transcription of over 1000 genes^11^. Emerging evidence has revealed that TYK2 promotes tumorigenesis, cell motility, and metastasis in several cancer types^12^. As a potential downstream target of TYK2, HSPA5 is a key regulator of protein folding and endoplasmic reticulum (ER) stress, which is often overexpressed in lung cancer, and is a promising biomarker and target for lung cancer^13,14^.

In this study, we confirmed that Rab27A functions as an oncogene in NSCLC cells. We also found that TYK2 is an important downstream protein of Rab27A whose phosphorylation causes a downstream cascade reaction by mediating the release of IFNα-containing exosomes, which results in the transcriptional activation of *HSPA5* to mediate the pathogenesis of NSCLC. These downstream effects were blocked by the TYK2 inhibitor cerdulatinib, while miR-124 inhibited the expression of *RAB27A* and, thus, Rab27A-mediated malignant biological behavior. Overall, our results confirmed that Rab27A activates IFNα-TYK2-HSPA5 signaling by promoting exosome secretion to regulate the pathogenesis of NSCLC and that its expression is negatively regulated by miR-124. These findings will provide important insights into the diagnosis and treatment of NSCLC.

## Results

### Rab27A is highly expressed in NSCLC tissues and cell lines and is associated with poor prognosis

**Fig. 1:**
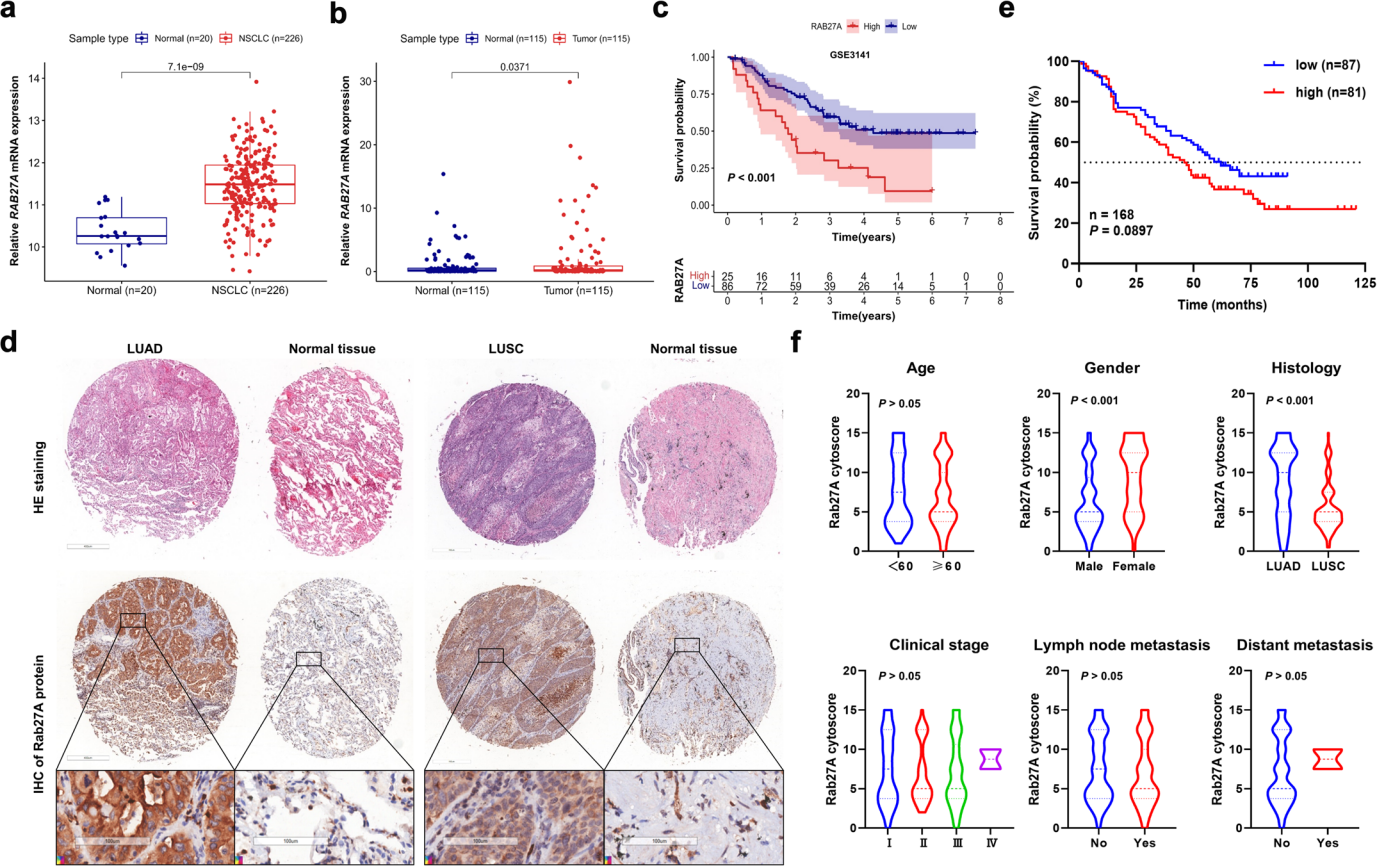
Overexpression of Rab27A in NSCLC and its effect on prognosis. **a** The expression data of *RAB27A* was downloaded from the GEO database (http://www.ncbi.nih.gov/geo) and was analyzed to compare the expression difference between NSCLC tissues (*N* = 226) and paired normal lung tissues (*N* = 20) (Microarray ID: GSE31210). **b** Relative *RAB27A* mRNA expression level of 115 NSCLC tissues and paired normal lung tissues. **c** Effect of the Rab27A expression level on the overall survival of NSCLC patients. The expression data and prognostic data were downloaded from the GEO database (Microarray ID: GSE3141). **d** Formalin-fixed and paraffin-embedded NSCLC tissues and paired normal lung tissues were performed IHC analyses of the Rab27A protein (Scale bar, 100 μm). **e** Survival analysis of patients in high score group (*N* = 81) and low score group (*N* = 87) according to the immunohistochemical score (cut-off value = 8). **f** Violin plots were generated to show the distribution of the Rab27A cytoscore across different clinical characteristics of patients. *P < 0.05; **P < 0.01; ***P < 0.001.

To explore the expression of Rab27A in NSCLC and its impact on patient prognosis, we performed statistical analyses using public databases and patient tissue samples. Based on published data from the gene expression omnibus (GEO) database, the mRNA expression level of *RAB27A* in NSCLC tissues was significantly higher compared with normal lung tissues (*P* < 0.001, Fig. 1a). In 115 pairs of clinical patient samples, the expression level of *RAB27A* in tumor tissues was significantly higher than that in paired normal tissues (*P* = 0.0371, Fig. 1b). Kaplan–Meier analysis revealed that patients with high *RAB27A* expression had significantly shorter OS (overall survival) (*P* < 0.001) than patients with low *RAB27A* expression (Fig. 1c). Moreover, in 168 NSCLC tissues and paired tumor-adjacent normal tissues, IHC analysis showed that the protein expression level of Rab27A was higher in NSCLC tissues than tumor-adjacent tissues (Fig. 1d). We divided the patients into high- and low-score groups according to the IHC score with a cut-off value = 5. Although there is no significant difference, the overall trend showed that patients in the high-score group had relatively shorter OS than patients in the low-score group (*P* = 0.0897, Fig. 1e). The expression of *RAB27A* was correlated with histological type and was higher in LUAD patients than in LUSC patients, which may be related to the differences in pathogenesis between LUAD and LUSC, and further exploration is needed (*P* < 0.001, Fig. 1f). Interestingly, the expression of *RAB27A* was correlated with gender (*P* < 0.001, Fig. 1f). These results confirmed that *RAB27A* was highly expressed in NSCLC and indicated the poor prognosis of patients.

### Effect of Rab27A on NSCLC cell proliferation and cell motility in vitro

**Fig. 2:**
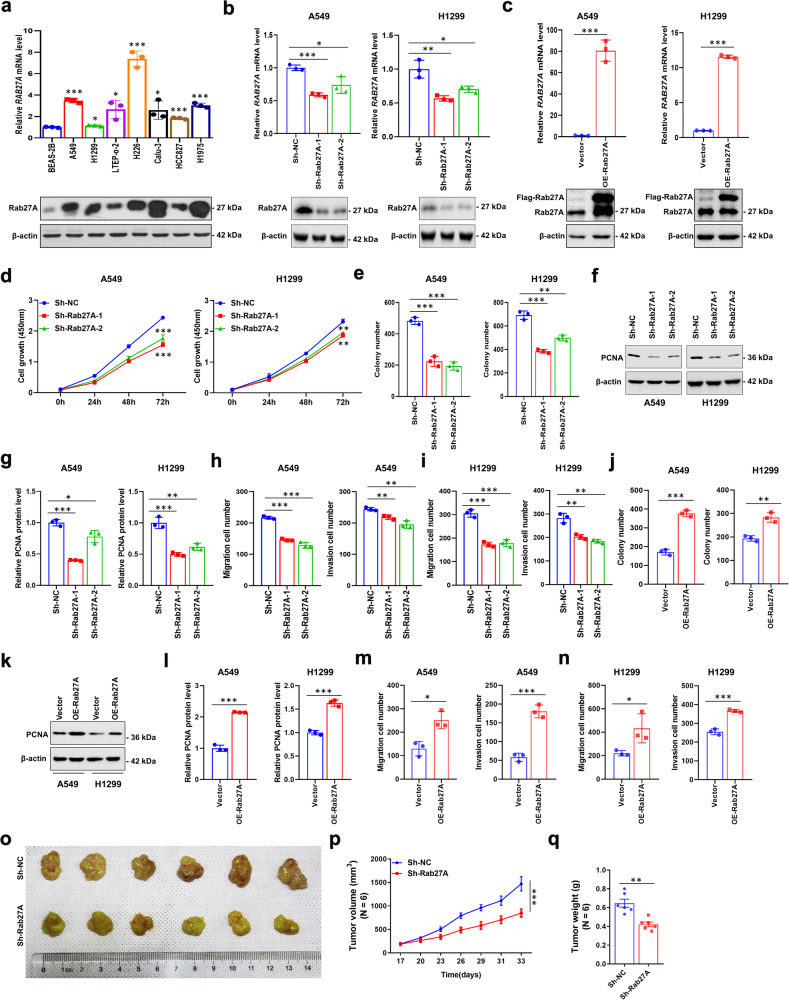
Inhibition of cell proliferation, migration, and invasion ability of NSCLC by knockdown of Rab27A. **a** The expression level of Rab27A in human NSCLC cells was detected by qRT-PCR and western blot. Data are shown as the mean ± SD of *n* = 3 technical replicates. **b** Rab27A mRNA and protein levels in Rab27A-knockdown NSCLC cells and negative control cells. **c** Rab27A mRNA and protein levels in Rab27A-overexpressing NSCLC cells and negative control cells. **d** CCK-8 assay to detect cell viability in A549 and H1299 cells; cell viability was determined at 24, 48, and 72 h. **e** Rab27A-knockdown inhibited the clonogenic ability of A549 and H1299 cells. **f**, **g** Cell lysates from A549 and H1299 cells infected with the indicated shRNAs were subjected to Western blot analysis to detect PCNA protein level. **h**, **i** Rab27A-knockdown inhibited the invasion and migration ability of A549 and H1299 cells. A549 and H1299 cells were allowed to migrate through an 8 μm pore Transwell. After 24 h, migrated cells were stained and counted in at least three microscopic fields (magnification, ×100). Then the cells were treated as above and allowed to invade through the Matrigel-coated membrane in Transwell. Invasive cells were stained and counted under a light microscope (magnification, ×100). **j** Rab27A-overexpression promoted the clonogenic ability of A549 and H1299 cells. **k**, **l** Cell lysates from A549 and H1299 cells with the indicated lentiviral vectors were subjected to Western blot analysis to detect PCNA protein level. **m**, **n** Rab27A-overexpression promoted the invasion and migration ability of A549 and H1299 cells. A549 and H1299 cells were allowed to migrate through an 8 μm pore Transwell. After 24 h, migrated cells were stained and counted in at least three microscopic fields (magnification, ×100). Then the cells were treated as above and allowed to invade through the Matrigel-coated membrane in Transwell. Invasive cells were stained and counted under a light microscope (magnification, ×100). **o** Rab27A-knockdown in A549 cells xenografts in nude mice (*n* = 6) at the experimental endpoint. Tumors were dissected and photographed as shown. **p** Tumor growth curves in mice (*n* = 6 in each group). **q** Each tumor formed was weighted. *P < 0.05; **P < 0.01; ***P < 0.001.

To further clarify the mechanism of Rab27A in mediating the pathogenesis of NSCLC, the regulatory effect of Rab27A on the proliferation, migration, and invasion of NSCLC cells was examined using in vitro experiments. qRT-PCR and western blotting analyses of the expression level of mRNA and protein of Rab27A in seven NSCLC cell lines and BEAS-2B cells showed that NSCLC cells have higher expression level of Rab27A than that in BEAS-2B cells (Fig. 2a). Immunofluorescence assays showed that Rab27A was mainly localized in the cytoplasm of NSCLC cells (Supplementary Fig. 1a). After the transfection with shRNAs to reduce RAB27A expression in A549 and H1299 cells, the mRNA and protein expression of Rab27A was significantly reduced compared to negative control cells (Fig. 2b). Accordingly, A549 and H1299 cells with *RAB27A* stably overexpression were constructed (Fig. 2c). CCK-8 assay showed that *RAB27A-*knockdown significantly inhibited the proliferation ability compared to control cells (Fig. 2d), while the clonogenic assay showed that the colony-forming ability of *RAB27A*-knockdown cells was significantly inhibited compared to that of the control cells (Fig. 2e, Supplementary Fig. 1b). In addition, the proportion of cells in the G0/G1 phase was significantly higher, while the proportion of cells in the S phase was significantly lower in *RAB27A*-knockdown cells than in control cells (Supplementary Fig. 1c). Western blotting showed that the expression of Cyclin D1, Cyclin A2, and PCNA in *RAB27A*-knockdown cells were significantly decreased compared with control cells (Fig. 2f, g, Supplementary Fig. 1d). Transwell assays using A549 and H1299 cells showed that the migration and invasion abilities of *RAB27A*-knockdown cells decreased significantly compared with those of control cells (Fig. 2h, i, Supplementary Fig. 1e). To clarify *RAB27A*-mediated tumor cell motility, we analyzed the expression of EMT (Epithelial-mesenchymal transition)-related markers. The mRNA and protein levels of MMP2, MMP9, N-cadherin, and Snail were significantly decreased in *RAB27A*-knockdown cells when compared with control cells (Supplementary Fig. 1f, g). In contrast, the clonogenic, migration, and invasion abilities increased significantly in *RAB27A*-overexpressing cells, and the expression levels of PCNA, MMP2, MMP9, N-cadherin, and Snail were increased in *RAB27A*-overexpressing cells when compared with control cells (Fig. 2j–n, Supplementary Fig. 1h–k). These results confirmed that Rab27A affects the proliferation, migration, and invasion of NSCLC cells in vitro.

### Knockdown of *RAB27A* can inhibit tumor growth in vivo

Based on the in vitro results, we further tested the regulatory effect of *RAB27A* knockdown on tumor growth in vivo. The stable *RAB27A*-knockdown A549 cell line was used to inoculate athymic BALB/C mice, and the tumors formed were significantly smaller in size than those formed by control cells, and the growth rates were significantly slower (Fig. 2o, p). Consistent with these results, tumor weight was significantly less in *RAB27A*-knockdown cell tumors than in the controls (Fig. 2q). In conclusion, these data confirmed that Rab27A expression can affect NSCLC growth in vivo.

### The secretion of exosomes in NSCLC cells was significantly affected by Rab27A

**Fig. 3:**
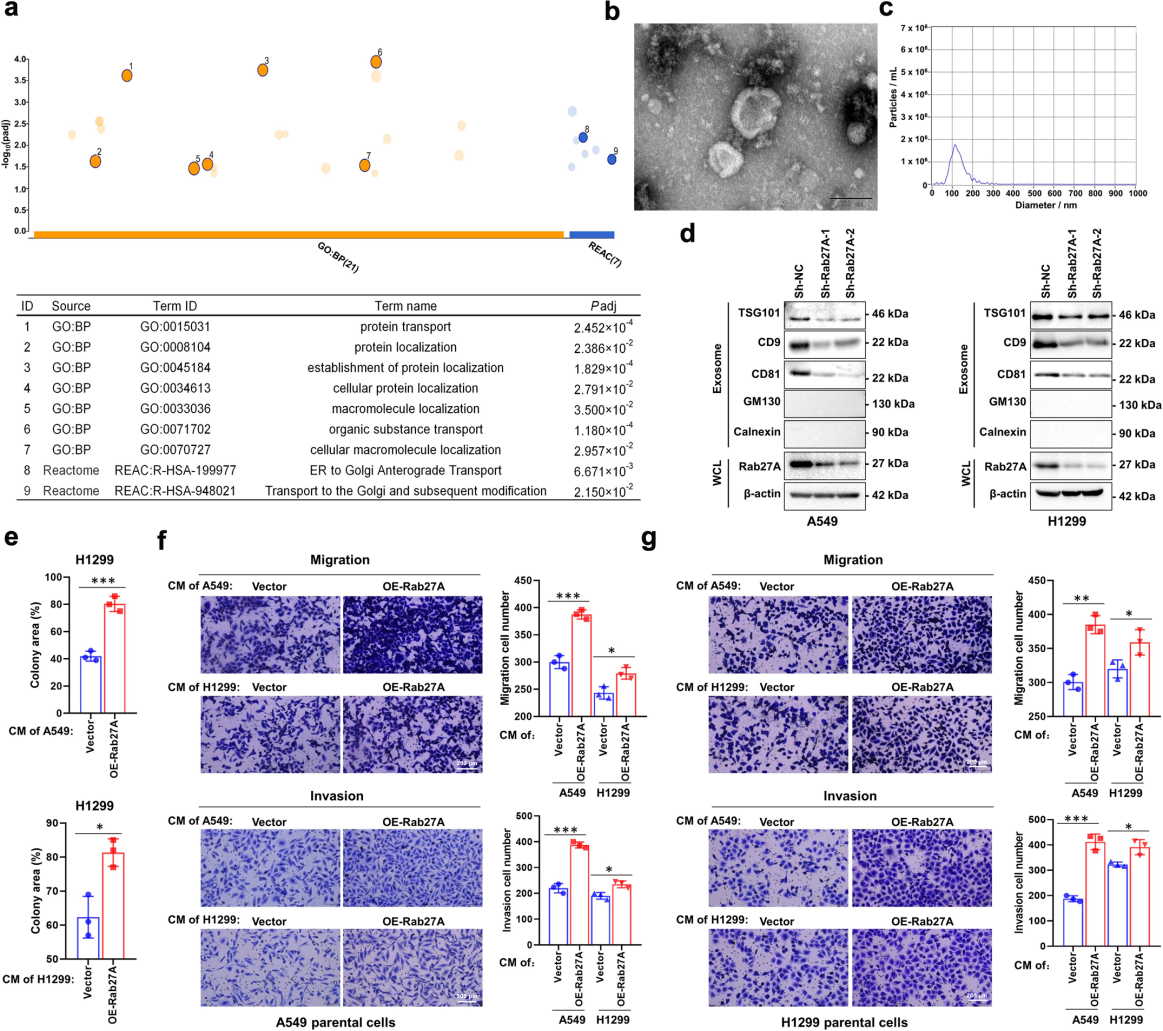
Effect of Rab27A on exosome secretion in NSCLC cells. **a** Functional enrichment analysis of differential protein showed that knockdown of Rab27A affects protein transport and protein localization function of NSCLC cells. **b** Transmission electron microscopy (TEM) image showed the appearance of exosomes isolated from NSCLC cell supernatant. **c** Nanoparticle tracking analysis (NTA) characterized the particle size distribution of isolated exosomes. **d** Western blot showed that the exosome markers TSG101, CD9, and CD81 in cell supernatant decreased significantly after the knockdown of Rab27A. GM130 and Calnexin were used as negative marker for exosome detection. **e** Conditioned medium (CM) collected from Rab27A-overexpression cells promoted the clonogenic ability of H1299 cells. **f** Conditioned medium (CM) collected from Rab27A-overexpression cells promoted the migration and invasion ability of A549 cells (Scale bar, 200 μm). **g** Conditioned medium (CM) collected from Rab27A-overexpression cells promoted the migration and invasion ability of H1299 cells (Scale bar, 200 μm). *P < 0.05; **P < 0.01; ***P < 0.001.

As an important protein mediating exosome secretion in cells, we analyzed whether *RAB27A* affects protein transport. Functional enrichment analysis based on protein mass spectrometry results showed that the function of Rab27A interacted proteins was mainly focused on protein transport and localization (Fig. 3a, Supplementary Table 1). Therefore, we investigated whether Rab27A affected exosome secretion in NSCLC cells. Isolated exosomes were processed and imaged using transmission electron microscopy (TEM), and characterized with nanoparticle tracking analysis (NTA), and used in subsequent Western blot and Transwell assay (Fig. 3b, c). Western blotting showed that after knockdown of *RAB27A* in A549 and H1299 cells, the exosome markers TSG101, CD9, and CD81 in the cell supernatant decreased significantly compared to that in control cells, suggesting that exosome secretion was inhibited (Fig. 3d). GM130 and Calnexin were used as negative marker for exosome detection (Fig. 3d). We further analyzed whether exosome secretion could affect the proliferation and migration of NSCLC cells by regulating the protein components in the cell culture supernatant. By collecting the cell culture supernatant from the stable cell line and stimulating the parent cell line, clonogenic assays, and Transwell assays confirmed that conditioned medium (CM) from *RAB27A* overexpression cells significantly upregulated the clonogenic growth, migration, and invasion abilities of NSCLC cells (Fig. 3e–g). Together, these results indicated that Rab27A could enhance NSCLC cell growth and migration by promoting exosome secretion.

### Rab27A promotes the phosphorylation of TYK2 in vitro

**Fig. 4:**
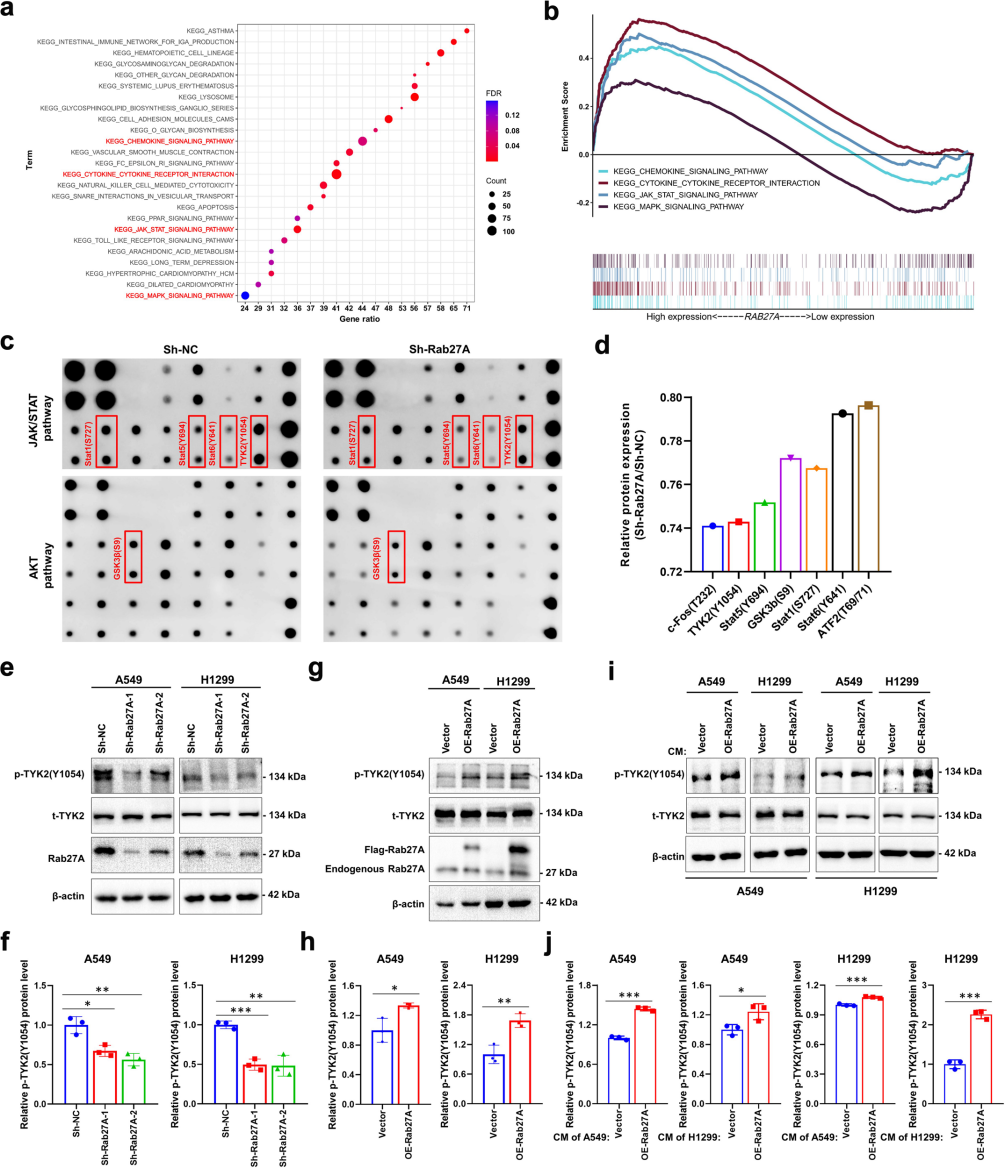
Rab27A activated the phosphorylation of TYK2. **a**, **b** GSEA based on TCGA data suggested that the high-level expression of Rab27A is related to the activation of the oncogenic pathway, chemokine pathway, JAK-STAT signaling pathway, MAPK signaling pathway, and TGF-β signaling pathway. **c** Plot map of phosphorylation pathway profiling array indicated the phosphorylation activation of JAK/STAT signaling pathway and AKT signaling pathway. **d** Relative protein expression level of Rab27A-knockdown cells and negative control cells based on phosphorylation pathway profiling array. **e**, **f** Cell lysates from A549 and H1299 cells with the indicated shRNAs were subjected to Western blot analysis to detect the phosphorylation activation of TYK2 (Y1054). **g**, **h** Cell lysates from A549 and H1299 cells with the indicated lentiviral vectors were subjected to Western blot analysis to detect the phosphorylation activation of TYK2 (Y1054). **i**, **j** After serum starvation for 24 h, A549, and H1299 cells were treated with CM collected from Rab27A-overexpressing cells or negative control cells for 24 h. Cell lysates were subjected to Western blot analysis to detect the phosphorylation activation of TYK2 (Y1054).

To further explore the regulatory mechanism of Rab27A in NSCLC, we analyzed the signaling pathways associated with *RAB27A* expression levels through gene set enrichment analysis (GSEA) based on the TCGA database. The results showed that high levels of *RAB27A* expression were closely related to the activation of the chemokine signaling, cytokine–cytokine receptor signaling, JAK-STAT signaling, and MAPK signaling pathways (*P* < 0.05, FDR < 0.25, Fig. 4a, b, Supplementary Fig. 2a). Furthermore, we analyzed the signaling pathway phosphorylation levels in *RAB27A* knockdown and control cells using a phosphorylation pathway profiling array, which showed that compared to the control cells, the TYK2 phosphorylation level decreased significantly in *RAB27A*-knockdown cell lines, suggesting that TYK2 phosphorylation may be monitored by Rab27A (Fig. 4c, d, Supplementary Fig. 2b–d, Supplementary Table 2). Moreover, *RAB27A* knockdown decreased TYK2 phosphorylation level, while *RAB27A* overexpression resulted in a significant increase in TYK2 phosphorylation (Fig. 4e–h). In addition, conditioned medium (CM) from *RAB27A* overexpression cells significantly increased TYK2 phosphorylation (Fig. 4i, j). These results indicated that Rab27A can affect the phosphorylation of TYK2 in NSCLC cells by affecting the components in the cell supernatant.

### Rab27A activates the IFNα-TYK2 axis by promoting exosome secretion

**Fig. 5:**
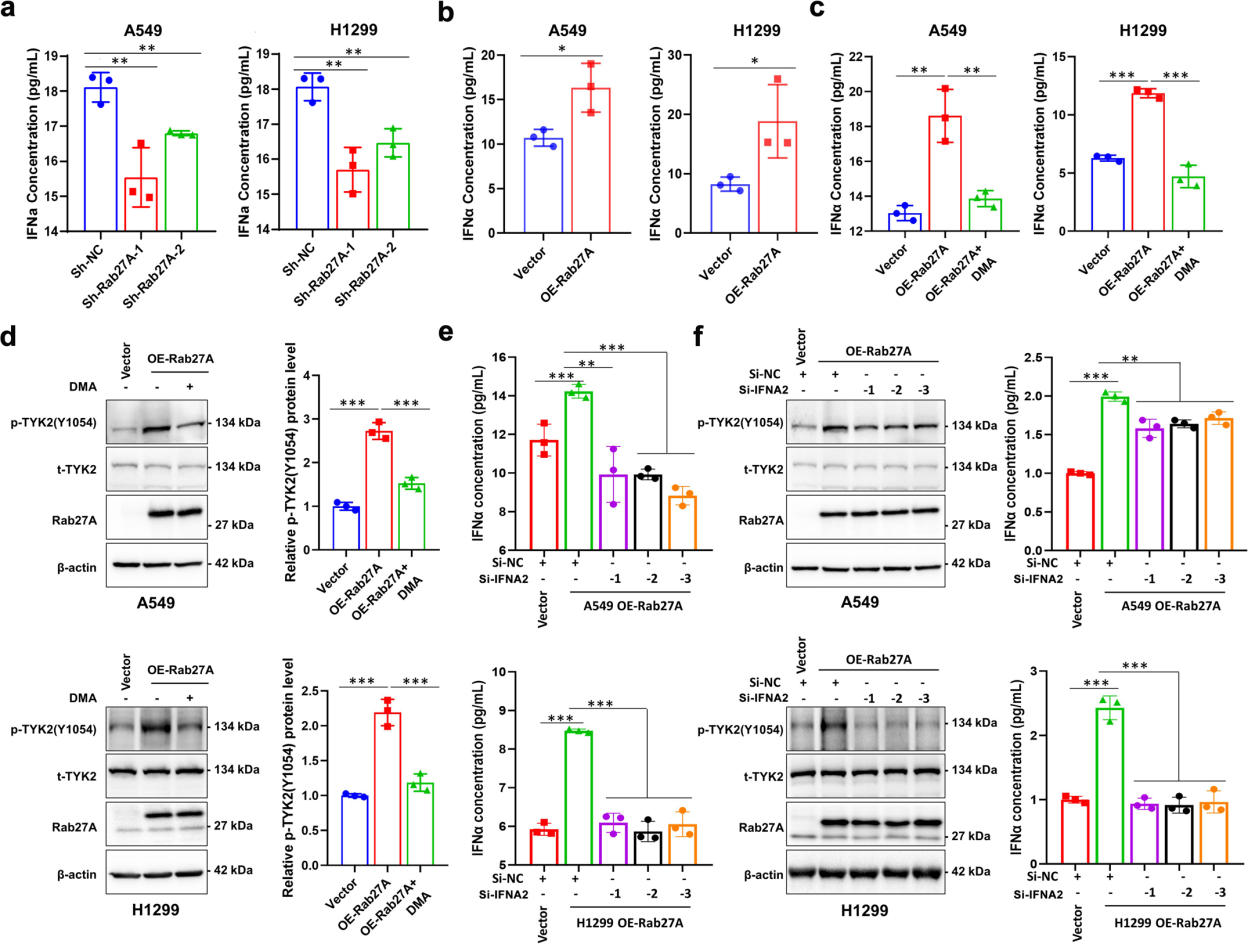
Rab27A promotes exosome-derived IFNα to upregulate TYK2 phosphorylation. **a** The IFNα concentration in the cell supernatant from A549 and H1299 cells with the indicated shRNAs. **b** The IFNα concentration in the cell supernatant from A549 and H1299 cells with the indicated lentiviral vectors. **c** Exosome secretion inhibitor DMA treatment significantly reduced the IFNα concentration in the cell supernatant of Rab27A-overexpressing A549 and H1299 cells. **d** The upregulation effect of TYK2 phosphorylation mediated by Rab27A-overexpression could be reversed by DMA in A549 and H1299 cells. **e** IFNA2-knockdown reversed the upregulation effect of IFNα concentration in the cell supernatant mediated by Rab27A-overexpression in A549 and H1299 cells. **f** The upregulation effect of TYK2 phosphorylation mediated by Rab27A-overexpression could be reversed by IFNA2-knockdown in A549 and H1299 cells.

Because the GSEA results indicated that Rab27A is associated with cytokine–cytokine receptor interaction and IFNα is one of the important ligands of TYK2, we analyzed the regulatory effect of Rab27A on IFNα-TYK2 signaling in NSCLC cells. Results from ELISA showed that the concentration of IFNα in the cell supernatant decreased or increased significantly when *RAB27A* was knocked down or overexpressed, respectively, when compared with the control cell supernatant (Fig. 5a, b). In addition, the cell supernatant content of IFNβ and IL-12, the other two important ligands of TYK2, were very low and were not significantly correlated with Rab27A expression (Supplementary Fig. 3a). We further confirmed that IFNα regulated the phosphorylation of TYK2 in A549 and H1299 cells in a time- and concentration-dependent manner (Supplementary Fig. 3b). Moreover, the concentration of IFNα in the cell supernatant decreased significantly after the addition of the exosome secretion inhibitor, DMA, suggesting that exosomes are an important source of IFNα in the cell supernatant (Fig. 5c, Supplementary Fig. 3c). In addition, the phosphorylation level of TYK2 upregulated by Rab27A-overexpression could be reversed by DMA treatment (Fig. 5d). After IFNα knockdown, the upregulation of IFNα concentration mediated by Rab27A-overexpression was reversed (Fig. 5e, Supplementary Fig. 3d). Consistently, the phosphorylation level of TYK2 upregulated by Rab27A-overexpression could be reversed by IFNα knockdown (Fig. 5f). These results confirmed that Rab27A promoted exosome secretion and increased IFNα in the cell supernatant to activate TYK2.

### Cerdulatinib reduces Rab27A-mediated biological functions through TYK2 inhibition

**Fig. 6:**
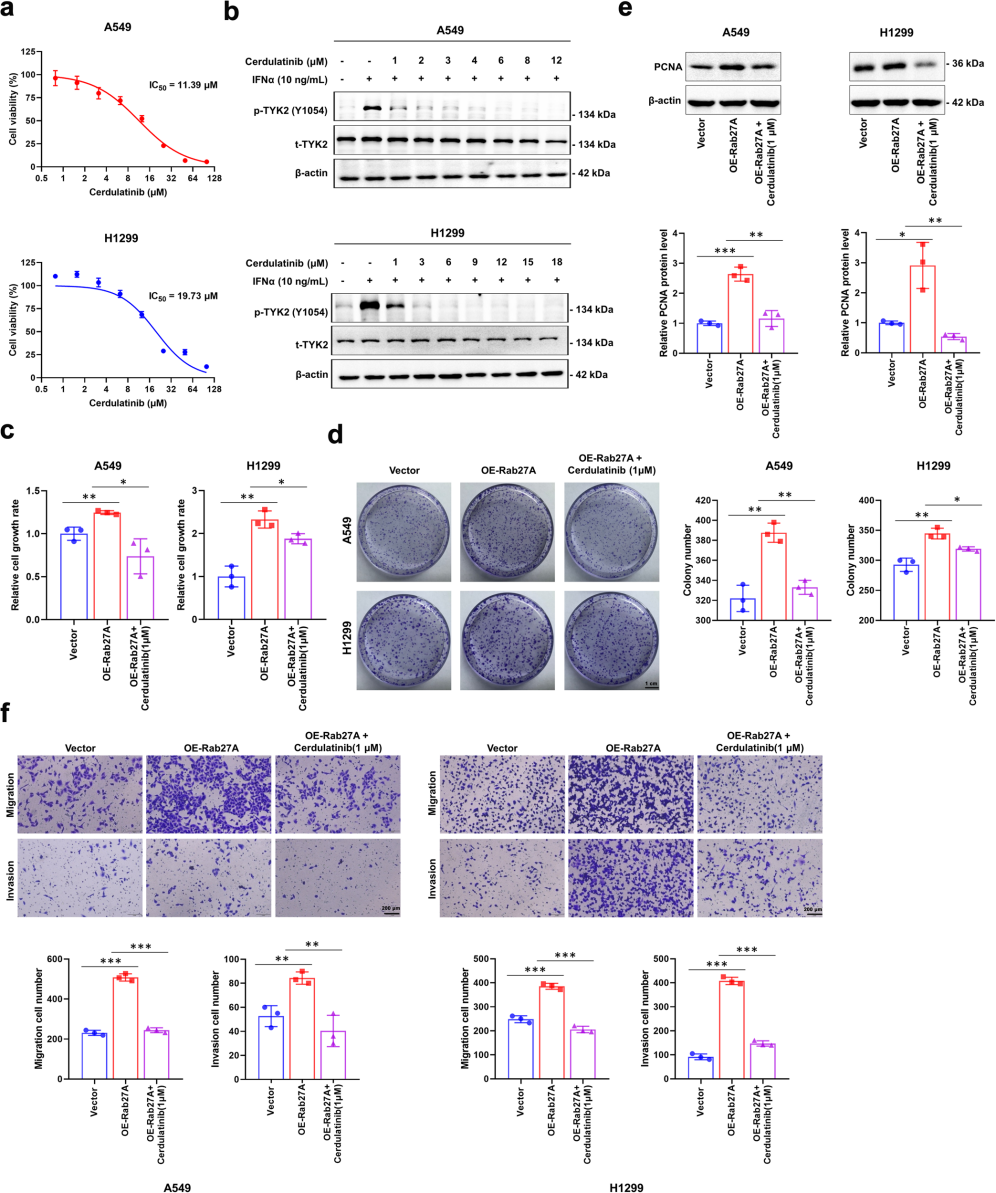
Pharmacological effects of Cerdulatinib on NSCLC cells. **a** The viability of A549 and H1299 cells was determined by CCK-8 assay after the cells treated with Cerdulatinib at the indicated concentration. The IC_50_ of Cerdulatinib for each cell line was calculated according to the cell viability value. **b** Cell lysates from A549 and H1299 cells treated with the indicated Cerdulatinib concentration were subjected to Western blot analysis to detect the phosphorylation activation of TYK2 (Y1054). **c** Cerdulatinib treatment (1 μM) reversed the promoting effect on cell growth ability induced by Rab27A-overexpression in A549 and H1299 cells. **d** Cerdulatinib treatment (1 μM) reversed the promoting effect on clonogenic ability induced by Rab27A-overexpression in A549 and H1299 cells (scale bar, 1 cm). **e** Cell lysates were subjected to Western blot analysis to detect the PCNA protein level. **f** Cerdulatinib treatment (1 μM) reversed the promoting effect on cell migration and invasion ability induced by Rab27A-overexpression in A549 and H1299 cells (Scale bar, 200 μm).

To determine the role of TYK2 in mediating the biological function of Rab27A, we tested the effect of the TYK2 inhibitor, cerdulatinib, on NSCLC cells. An in vitro cytotoxicity assay showed that cerdulatinib had a significant inhibitory effect on the cell viability of A549 and H1299 cells (IC50_A549_ = 11.39 μM, IC50_H1299_ = 19.73 μM, Fig. 6a, Supplementary Fig. 3e) and western blotting confirmed that cerdulatinib significantly inhibited the phosphorylation of TYK2 in A549 and H1299 cells (Fig. 6b). The CCK-8 assay showed that *RAB27A* overexpression significantly enhanced the proliferation of cells compared with control cells, and significantly inhibited after treatment with 1 μM cerdulatinib (Fig. 6c). Moreover, the clonogenic assay showed that *RAB27A* overexpression significantly enhanced the colony-forming ability of cells compared with that of control cells, and this promotion trend was significantly inhibited by cerdulatinib treatment (Fig. 6d). Western blotting analysis showed that the expression of PCNA was significantly increased in *RAB27A*-overexpressing cells when compared with that in control cells, and this increase was significantly inhibited by cerdulatinib treatment (Fig. 6e). Transwell assays showed that *RAB27A* overexpression significantly enhanced the migration and invasion ability of cells compared with control cells, and this promotion trend was significantly inhibited by cerdulatinib treatment (Fig. 6f). qRT-PCR analysis showed that the expression of the EMT markers (MMP2, MMP9, and N-cadherin) was significantly increased in *RAB27A*-overexpressing cells when compared with that in control cells, and this increase was significantly inhibited by cerdulatinib treatment (Supplementary Fig. 3f). These results suggested that Rab27A can promote cell proliferation, migration, and invasion by regulating IFNα-TYK2 signaling, and this promotion can be inhibited through the inhibition of TYK2 activity.

### *HSPA5* is an important downstream target of the IFNα-TYK2 axis signaling

**Fig. 7:**
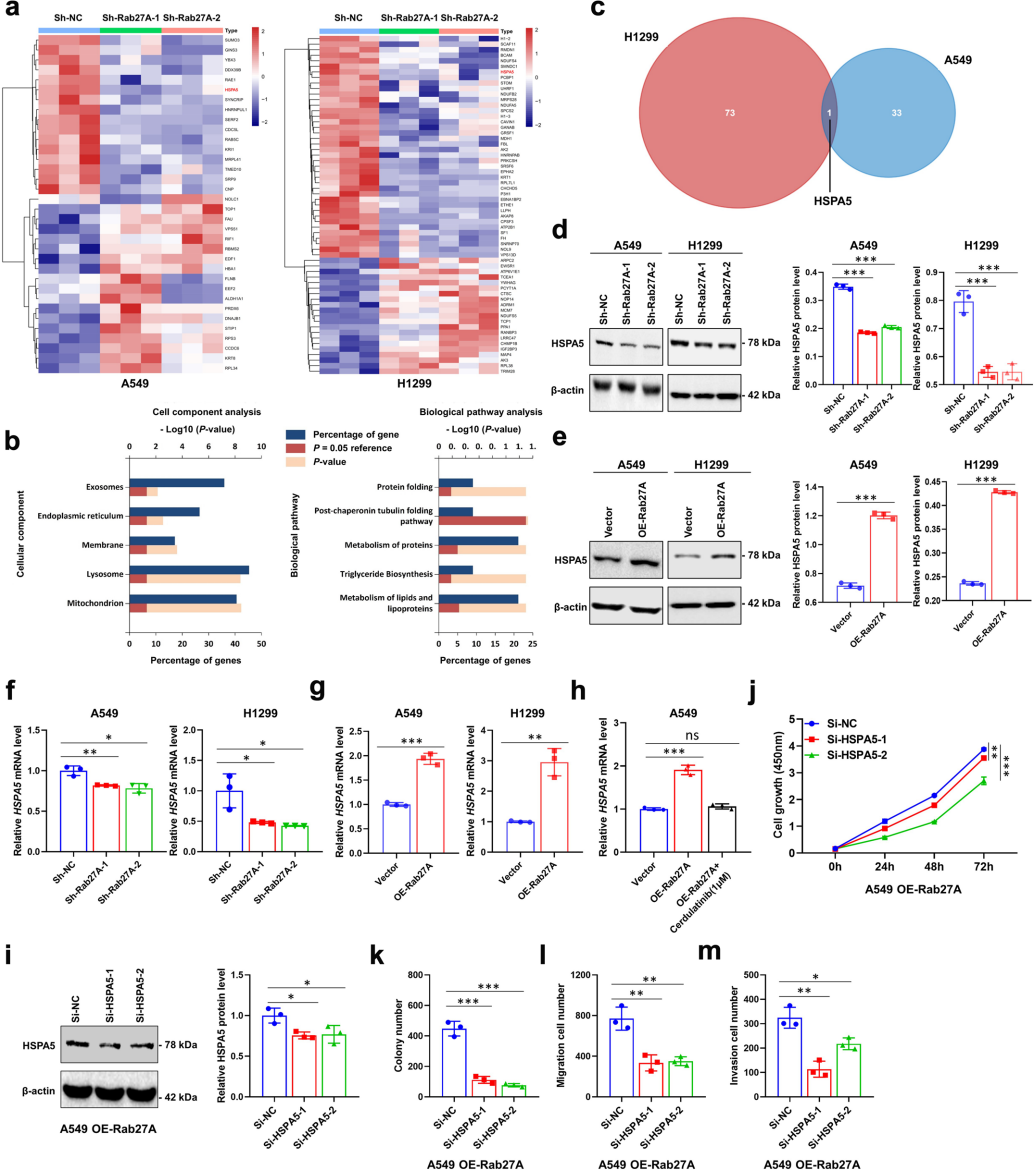
HSPA5 expression is affected by Rab27A and ER stress may be the potential downstream mechanism to mediate malignant biological behavior. **a** Heatmap of differentially expressed protein in A549 and H1299 cells with Rab27A-knockdown. **b** GO analysis based on differentially expressed protein showed the potential regulated cellular components and biological pathways. **c** Venn diagram showed the intersection of differentially expressed protein between A549 and H1299 cells. **d**, **e** Cell lysates from A549 and H1299 cells infected with the indicated shRNAs or lentiviral vectors were subjected to Western blot analysis to detect the HSPA5 protein level. **f**, **g** qRT-PCR analysis of *HSPA5* mRNA in A549 and H1299 cells infected with the indicated shRNAs or lentiviral vectors. **h** Cerdulatinib treatment (1 μM) reversed the promoting effect on *HSPA5* mRNA expression induced by Rab27A overexpression. **i** SiRNAs targeting HSPA5 assay was performed in A549 OE-Rab27A cells for 48 h. Cell lysates were subjected to Western blot analysis to detect the HSPA5 protein level. **j** The viability of A549 cells overexpressing Rab27A was determined by CCK-8 assay after the knockdown of HSPA5. **k–m** Rab27A-knockdown inhibited the clonogenic ability, migration ability, and invasion ability of A549 cells overexpressing Rab27A.

To further explore the molecular mechanism of Rab27A-mediated malignant biological behavior in NSCLC, used protein mass spectrometry to measure changes in protein composition with or without *RAB27A* expression. The results showed that knockdown of *RAB27A* caused several changes in the protein in A549 and H1299 cells when compared with that of control cells (Fig. 7a). Gene ontology (GO) enrichment analysis showed that the differentially expressed proteins were closely related to several cellular components, including exosomes and the endoplasmic reticulum, and were involved in the protein folding pathway (Fig. 7b). A Venn diagram was used to illustrate that HSPA5 was downregulated in A549 and H1299 cells with *RAB27A*-knockdown (Fig. 7c). Moreover, synchronous changes in *HSPA5* mRNA and protein levels were also observed in *RAB27A* stably-transfected cells, suggesting that *HSPA5* transcription was regulated by Rab27A in these cells (Fig. 7d–g). In cell lines overexpressing *RAB27A*, *HSPA5* transcription was activated, and this effect was reversed by cerdulatinib (Fig. 7h). After the knockdown of *HSPA5* due to the overexpression of *RAB27A*, cell growth, clonogenic ability, migration ability, and invasion ability were inhibited (Fig. 7i–m, Supplementary Fig. 4). These results suggest that Rab27A may mediate the malignant biological behavior of NSCLC cells by regulating the transcription of *HSPA5*, and this activation can be reversed by cerdulatinib.

### STAT1 activates *HSPA5* transcription

**Fig. 8:**
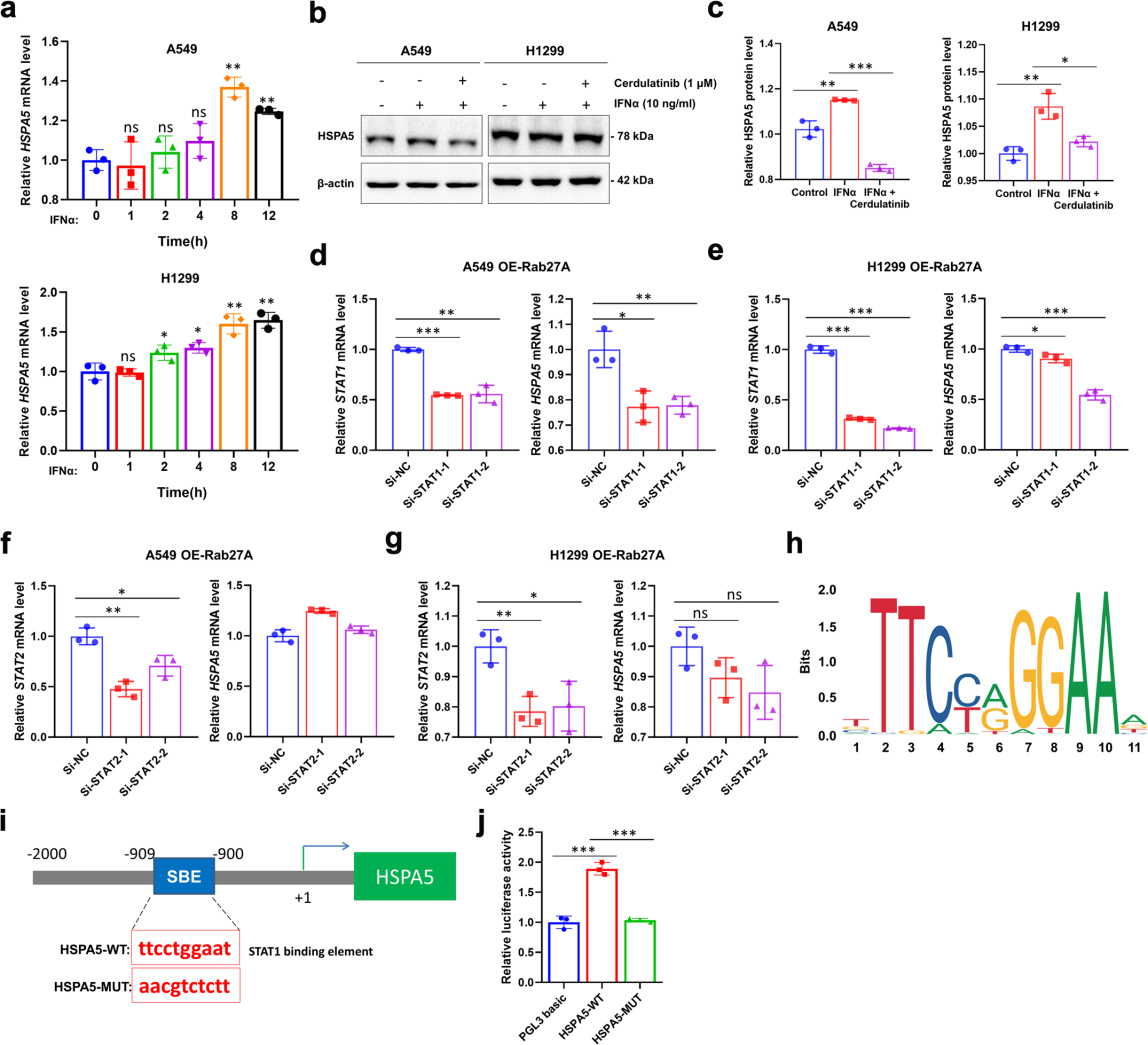
STAT1 transcriptionally regulates *HSPA5* gene in LUADs. **a** qRT-PCR analysis of *HSPA5* mRNA in A549 and H1299 cells treated with IFNα at the indicated time. **b,** **c** Cell lysates from A549 and H1299 cells treated with Cerdulatinib and IFNα were subjected to Western blot analysis to detect the expression of HSPA5. **d**, **e** qRT-PCR analysis of *STAT1* mRNA in A549 and H1299 cells with Rab27A-overexpression after *STAT1*-silencing. **f**, **g** qRT-PCR analysis of *STAT2* mRNA in A549 and H1299 cells with Rab27A-overexpression after *STAT2*-silencing. **h** A conserved consensus *HSPA5* response element was present. **i** Schematic drawing shows a predicted SBE in *HSPA5* mRNA 3’UTR. Boxed areas indicate the *HSPA5* 3’UTR segment (position, −909 to −900), containing wild type (*HSPA5*-WT) or mutant of SBE (*HSPA5*-MUT). Numbers present the nucleotide position of *HSPA5* mRNA. **j** Relative luciferase activities were determined as described in the “Methods” section and normalized to those in vector cells.

We next explored why *HSPA5* was upregulated by *RAB27A* overexpression. First, after A549 and H1299 cells were treated with IFNα, the mRNA level of *HSPA5* gradually increased and peaking at 8–12 h (Fig. 8a). Furthermore, the stimulatory effect of IFNα on *HSPA5* transcription could be inhibited with cerdulatinib treatment (Fig. 8b, c). Previous studies have shown that the STAT1/STAT2 complex is an important downstream transcription factor of the IFNα-TYK2 signaling pathway; thus, we constructed STAT1- or STAT2-silencing in stable *RAB27A*-overexpression A549 and H1299 cell lines. The results showed that after STAT1-silencing, *HSPA5* mRNA expression was inhibited, while STAT2-silencing did not significantly inhibit *HSPA5* mRNA expression (Fig. 8d–g). In the STAT1/STAT2 complex, STAT1 is mainly responsible for DNA binding, whereas STAT2 does not interact directly with DNA but has a transcription activation domain. Therefore, we identified the position of the functional STAT1-binding site and confirmed that it was localized at positions −909 to −900 in the *HSPA5* gene (Fig. 8h–j). These results showed that *HSPA5* transcription was activated by STAT1.

### Cerdulatinib plays an anti-tumor role in vivo

**Fig. 9:**
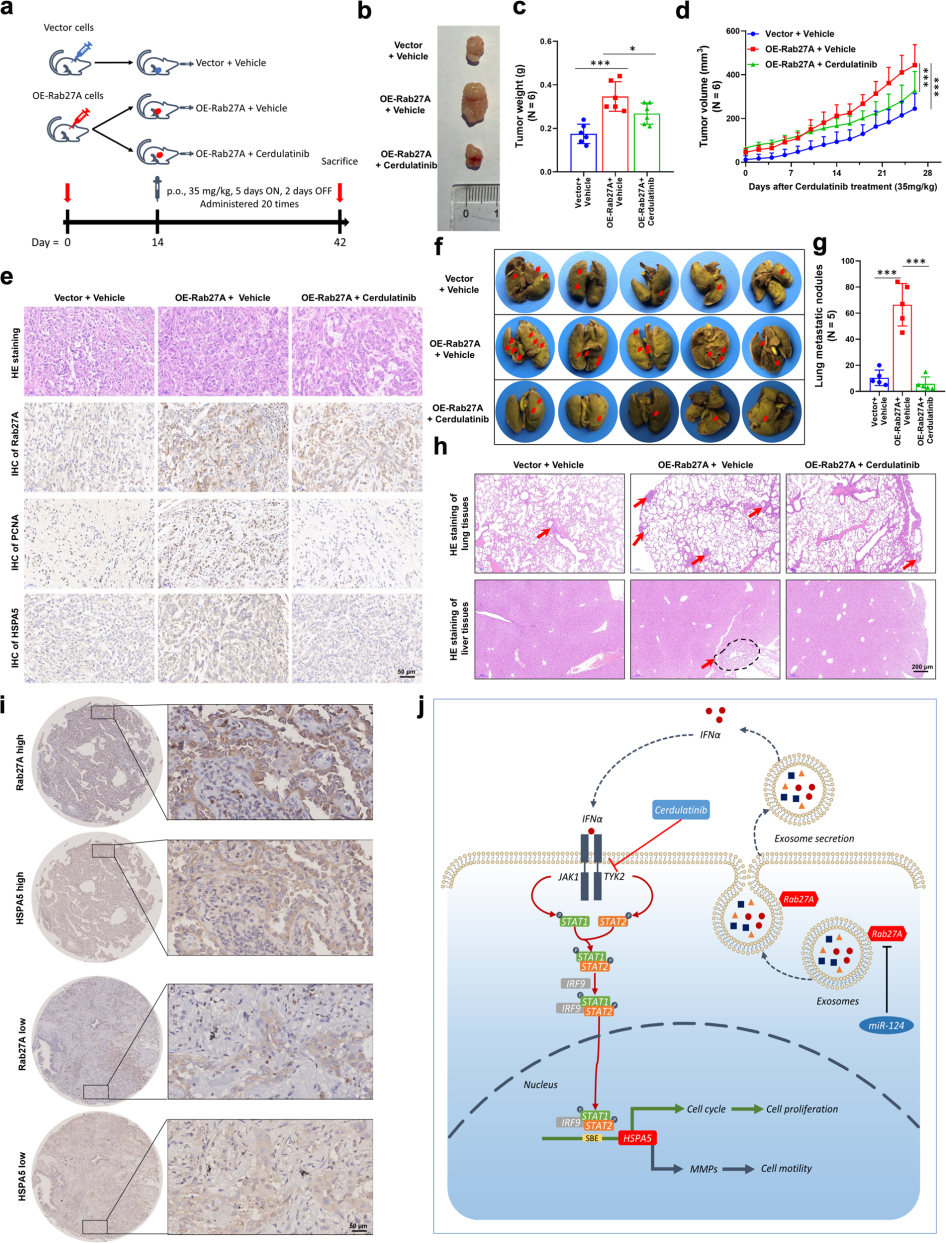
Cerdulatinib alleviates Rab27A-induced malignant progression of NSCLC. **a** Schematic flowchart of A549 cell in vivo xenograft model. Rab27A-overexpression and control vector A549 cells (3 × 10^6^ cells/mouse) were injected into BALB/c nude mice (*N* = 6 mice per group). Cerdulatinib (35 mg/kg) was administrated p.o. for 20 times (5 days on, 2 days off). **b** A549 cell xenografts in nude mice (*N* = 6 mice per group) at the experimental endpoint; tumors were dissected and photographed as shown. **c** Each tumor formed was weighted. **d** Tumor growth curves in mice (*N* = 6 mice per group) inoculated with the indicated cells on the indicated days. **e** Hematoxylin and eosin (H&E) staining confirmed the presence of tumor cells in the indicated tumor sections. Immunohistochemical staining for Rab27A, PCNA, and HSPA5 was performed (scale bar, 50 μm). **f** Representative images of lung metastatic nodules developed in mice 6 weeks after injection of Rab27A-overexpressing A549 cells or control A549 cells. The surgically resected lungs were stained as described in the “Methods” section. Red arrowheads indicate metastatic nodules established in lungs. **g** Comparison of the number of lung metastatic nodules between Rab27A-overexpressing group and vector group (*N* = 5 mice per group). Data are shown as the boxplot. **h** H&E staining was performed for the evaluation of lung micro-metastases. Representative images showing micro-metastases of lung tissues from a pair of mice referred in (**f**). Red arrowheads indicate lung micro-metastases of vector group and Rab27A-overexpressing group, respectively (scale bar, 200 μm). **i** Formalin-fixed and paraffin-embedded NSCLC tissues and paired normal lung tissues were performed IHC analyses of the HSPA5 and Rab27A protein. **j** A proposed work model: Rab27A activates IFNα-TYK2-HSPA5 signaling via regulating exosome secretion to mediate pathogenesis and progression of non-small cell lung cancer. *P < 0.05; **P < 0.01; ***P < 0.001.

To further elucidate the antitumor effect of cerdulatinib on NSCLC cells in vivo, we injected BALB/C nude mice inoculated with A549 cells stably overexpressing *RAB27A* and vector control A549 cells (Fig. 9a). At 2 weeks post-inoculation, the mice that were injected with *RAB27A*-overexpressing A549 cells were divided into two groups to receive 35 mg/kg cerdulatinib or vehicle. Cerdulatinib treatment suppressed the tumor-promoting effect of *RAB27A*-overexpression when compared to the vehicle group (Fig. 9b–d). Additionally, H&E staining of tumor sections confirmed the presence of tumor cells (Fig. 9e). IHC staining for Rab27A, PCNA, and HSPA5 was quantified based on the staining intensity and indicated that the protein level of Rab27A, PCNA, and HSPA5 was increased in *RAB27A*-overexpressing tumors, and decreased after cerdulatinib treatment (Fig. 9e). BALB/C nude mice were intravenously injected with A549 cells stably overexpressing *RAB27A* and vector control A549 cells, followed by treatment with 35 mg/kg cerdulatinib. We found that mice injected with *RAB27A*-overexpressing A549 cells developed more metastatic lung nodules than those injected with control A549 cells, and this Rab27A-promoted metastasis was inhibited by oral administration of cerdulatinib (Fig. 9f, g). The results of H&E staining showed that the injection of *RAB27A*-overexpressing A549 cells resulted in more micro-metastatic foci in lung and liver tissues than in the vector group, and the tumor-promoting effect of *RAB27A*-overexpression could be reversed with cerdulatinib treatment (Fig. 9h). Moreover, formalin-fixed and paraffin-embedded NSCLC tissues and paired normal lung tissues were performed IHC to analyses the correlation between HSPA5 and Rab27A protein (Fig. 9i). Taken together, the in vivo metastasis assays showed that cerdulatinib can reverse the tumor proliferation mediated by overexpression of *RAB27A* and plays an antitumor role in vivo.

### Co-expression pattern between *HSPA5* and *RAB27A*

To further investigate the correlation between HSPA5 and Rab27A, we analyzed whether HSPA5 and Rab27A were related to the expression level of mRNA using public databases. The mRNA levels of *HSPA5* were upregulated in several cancer types (Supplementary Fig. 5a), and the GEO database showed that the mRNA expression level of *HSPA5* in NSCLC tissues was significantly higher than that in normal lung tissues (*P* < 0.001, Supplementary Fig. 5b). IHC showed that HSPA5 was highly expressed in lung tumor tissues compared to that in paired normal tissues in patients (Supplementary Fig. 5c, *P* < 0.001). Based on Kaplan–Meier analysis of survival probability, it was found that patients with high *HSPA5* expression had significantly shorter OS (*P* < 0.001) than patients with low *HSPA5* expression (Supplementary Fig. 5d). Moreover, *HSPA5* and *RAB27A* mRNA were co-expressed in NSCLC tissues (Supplementary Fig. 5e). Formalin-fixed and paraffin-embedded human NSCLC tissues and paired normal lung tissues were subjected to IHC to visualize the expression of HSPA5 and Rab27A proteins (Fig. 9i). Further analysis showed that HSPA5 was significantly expressed in tumor tissues and co-expressed with Rab27A protein (Supplementary Fig. 5f). Taken together, these results indicated that HSPA5 and RAB27A are co-expressed in NSCLC cells.

### miR-124 can inhibit *RAB27A* expression

To further clarify the expression regulation mechanism of Rab27A, miRNAs that bind to the 3’-UTR of *RAB27A* mRNA were analyzed using bioinformatic tools. The results showed that miR-124 could bind to the 3’-UTR of *RAB27A* mRNA, and high miR-124 expression predicted a better prognosis for NSCLC patients (Supplementary Fig. 6a, b). In addition, transfection of miR-124 mimics reduced Rab27A mRNA and protein levels in A549, H1299, H226 cells, whereas transfection of an miR-124 inhibitor increased *RAB27A* mRNA expression, which confirmed that *RAB27A* was negatively regulated by miR-124 (Supplementary Fig. 6c–e). A dual luciferase reporter (DLR) assay confirmed that miR-124 could bind to the 3’-UTR of *RAB27A* mRNA in A549, H1299, and H226 cells (Supplementary Fig. 6f). To further analyze the effect of miR-124 on NSCLC cells in vivo, A549 cells transfected with miR-124 agomir was injected into athymic BALB/c mice. The tumors formed by the cells transfected with the miR-124 agomir were significantly smaller in size than those formed by control cells, and the tumor growth rate was slower (Supplementary Fig. 6g). The resected xenograft tumor tissues were analyzed using IHC and qRT-PCR to verify the expression of Rab27A. The results showed that the expression level of Rab27A was significantly lower in tumors formed by cells transfected with the miR-124 agomir than in the control tumors (Supplementary Fig. 6h, i). Consistently, the tumor weight was significantly lower in cells transfected with the miR-124 agomir, and the tumor growth rate was significantly slower than those of the negative controls (Supplementary Fig. 6j, k). Clonogenic and transwell assays showed that the colony formation, migration, and invasion abilities of A549 cells decreased after transfection with miR-124 mimics, and *RAB27A*-overexpression reversed this inhibition (Supplementary Fig. 6l, m). Furthermore, IFNα concentration decreased in H1299 *RAB27A*-overexpressing cells after transfection with miR-124 mimics (Supplementary Fig. 6n). In conclusion, in vitro and in vivo experiments confirmed that the expression of *RAB27A* was negatively regulated by miR-124 and that the malignant behavior mediated by Rab27A was suppressed by miR-124.

## Discussion

The treatment of NSCLC remains a global challenge and understanding the mechanism underlying the occurrence and development of NSCLC is urgent to better identify reliable therapeutic targets^2^. As an important exosome transport-related protein, the oncogenic function of Rab27A has been confirmed in several cancer types^15–19^. However, the role and underlying mechanism of Rab27A in NSCLC remain to be elucidated.

Exosomes are discoid vesicles containing complex RNAs and proteins with a diameter of 50–100 nm and involved in signaling communication between cells^20–22^. Because Rab27A is responsible for the formation and secretion of exosomes, a comprehensive study of its function would help the development of minimally invasive diagnosis techniques^23,24^.

This presented study reports that the molecular mechanism of Rab27A is an important exosome transporter in promoting tumorigenesis. Results indicate that Rab27A plays an important role in the oncogenesis of NSCLC cells. Rab27A affects the IFNα-TYK2 signaling axis by regulating the secretion of exosomes and causing the transcriptional activation of *HSPA5*to mediate a series of malignant biological behaviors in NSCLC. This study also revealed the significance of the TYK2 inhibitor cerdulatinib in the treatment of NSCLC. These findings provide insights and therapeutic tactics for the clinical NSCLC treatment.

Consistent with previous studies, we proved that *RAB27A* is an oncogene in NSCLC that can promote the proliferation and motility of NSCLC cells in vitro^15–17^. Interestingly, the expression level of *RAB27A* is associated with gender in NSCLC patients, we speculate that this may be due to its expression being correlated with the estrogen level or having a mutually regulatory relationship with genes related to gender. Of course, more data validation is needed to support this conjecture. In vivo experiments confirmed that Rab27A promoted tumor growth, and we further explored the possible downstream signaling pathway affected by Rab27A using a Phosphorylation Pathway Profiling Array to show that IFNα-TYK2 signaling is monitored by *RAB27A* expression and mediated through exosome secretion. We showed that the HSPA5 protein was affected by Rab27A. As a key molecule in the unfolded protein response (UPR) pathway, the transcriptional activation of *HSPA5* is involved in mediating the malignant biological behavior of cancer cells^13,25–28^. Therefore, we hypothesized that Rab27A activates IFNα-TYK2 signaling by regulating exosome secretion and mediating *HSPA5* transcriptional activation to promote malignant biological behavior in NSCLC. Our other experiments confirmed that upregulation of the STAT1/2 complex was important for the activation of *HSPA5* transcription. As previous studies have indicated, the transcriptional activation effect of STAT2 requires STAT1 to maintain a stable interaction with DNA^29,30^. In addition, miR-124 is an important miRNA inhibiting the expression of *RAB27A*.

The protein encoded by *HSPA5* is HSP70, a member of the heat shock protein 70 family^31,32^. HSPA5 localizes to the lumen of the ER, where it participates in the folding and assembly of proteins^33,34^. Because HSPA5 plays an important role in monitoring intracellular protein trafficking by interacting with ER proteins, it^13,14^. We confirmed that *HSPA5* expression is regulated by Rab27A and showed that the IFNα-TYK2 signaling activation by Rab27A can induce the nuclear entry of downstream transcription factors and activate the transcription of *HSPA5* to mediate carcinogenesis associated with NSCLC.

Cerdulatinib was shown to have extensive clinical activity in B- and T-cell malignancies; however, its pharmacological activity in solid tumors has not been fully studied^35–37^. We found that cerdulatinib had an obvious growth inhibitory effect on NSCLC and could reverse the proliferation effect caused by *RAB27A* overexpression. A xenograft mouse model confirmed that cerdulatinib could significantly inhibit tumor growth in vivo and reduce the tumor growth-promoting effect caused by the *RAB27A* overexpression.

Overall, our study reported that the overexpression of *RAB27A* activates IFNα-TYK2-STAT signaling by promoting exosome secretion, which leads to the transcriptional activation of *HSPA5*, and finally mediates a series of malignant biological behaviors characteristic of NSCLC. Furthermore, we found that miR-124 can inhibit the expression of *RAB27A*, thereby reducing cell proliferation and motility in vitro and in vivo. Our findings reveal the function of Rab27A in the occurrence and development of NSCLC through regulation of the IFNα-TYK2 signaling axis (Fig. 9j). These results provide insights into the study of exosome secretion in NSCLC and promising strategies for future clinical practice.

## Methods

### NSCLC tissue samples

Paired NSCLC tissue and adjacent noncancerous lung tissue samples (115 of each) were collected with informed consent from patients at the First Affiliated Hospital of Soochow University between 2015 and 2018. The patients were diagnosed with NSCLC based on their histological and pathological characteristics. None of the included patients had undergone chemotherapy or radiotherapy prior to the tissue sampling. The tissue samples were snap-frozen and stored in a cryofreezer at −80 °C. All research protocols were approved by the Ethics Committee of the First Affiliated Hospital of Soochow University (approval no. 2018-255) and conducted in accordance with all relevant ethical regulations, including the Declaration of Helsinki. Written informed consent to participate in this study has been provided from all patients.

### Cell lines and cell culture

The human embryonic kidney (HEK) 293T, human immortalized bronchial epithelial BEAS-2B, and NSCLC H1299, A549, LTEP-α-2, H226, Calu-3, HCC827, and H1975 cell lines were purchased from Procell Life Science & Technology Co., Ltd. (Wuhan, China). The cells were cultured in DMEM high-glucose medium or RPMI-1640 medium (HyClone, South Logan, UT, USA) containing 10% fetal bovine serum (FBS; Gibco, Carlsbad, CA, USA) and 1% penicillin-streptomycin (Beyotime, Shanghai, China) in a humidified atmosphere with 5.0% CO_2_ at 37 °C. The cell supplier had confirmed the genetic characteristics of these cells. All these cell lines were passaged within 6 months.

### Plasmid constructs, production of lentivirus, and cell transduction

To construct stable *RAB27A*-overexpressing A549 and H1299 cell lines, the coding sequences of *RAB27A* (GenBank Accession number: NM_004580.5) were amplified using specific primers with the KOD Plus Mutagenesis Kit (TOYOBO) and subcloned into the pCDH-CMV-MCS-EF1-copGFP lentiviral vector downstream of the CMV promoter using the restriction endonucleases NheI and BamHI. To establish stable *RAB27A*- silenced cell lines, two targeted *RAB27A* shRNA fragments were subcloned into the pGMLV-SC5 lentiviral vector (Genomeditech, Shanghai, China) using the endonucleases BamHI and EcoRI (Fermentas). All the constructed plasmids were sequenced by GENEWIZ Biotechnology (Suzhou, China) before use. The constructed plasmids were co-transfected into HEK293T cells with viral packaging plasmids VSVG, REV, and MDL using PEI (Polysciences). The corresponding empty vectors served as negative controls. After 48 h, the cell supernatant was filtered with a 0.22 μm strainer and used to infect A549 and H1299 cells. Finally, stably overexpressing *RAB27A* cells were selected by fluorescence-activated cell sorting (FACS), and stably silenced *RAB27A* cells were filtered with 2.0 μg/mL puromycin (Beyotime, Shanghai, China). The primer sequences used for constructing *RAB27A* plasmids are listed in Supplementary Table 3.

### RNA interference

Two small interfering RNA (siRNA) sequences targeting coding regions of *RAB27A/STAT1/STAT2* were synthesized by Gene Pharma company (Suzhou, China). Scrambled siRNA was served as the negative control (si-NC). A549 and H1299 cells were seeded into six-well cell plates and transiently transfected with 100 pmol of siRNA using the transfection reagent Lipofectamine 2000 (Invitrogen, Waltham, MA, USA). After 72 h of transfection, cells were harvested for subsequent assays. The Si-NC and target sequences of the siRNAs are listed in Supplementary Table 4.

### RNA extraction, cDNA synthesis, and quantitative real-time PCR (qRT-PCR) analysis

Cells or tissues were homogenized in TRIzol Reagent (Takara, Osaka, Japan) and total RNA extraction according to the standard protocol. The concentration of RNA was measured using a NanoDrop 2000 (Thermo Fisher Scientific, Waltham, MA, USA). The cDNA synthesis was performed using M-MLV reverse transcriptase (Takara), and qRT-PCR was performed with SYBR Premix ExTaq^TM^ (Takara) using ABI Step One Plus Real-Time PCR system (Applied Biosystems, Foster City, CA, USA). The PCR program was as follows: 95 °C for 10 min, followed by 40 cycles of 95 °C for 15 s, and 60 °C for 1 min. The Ct values of *RAB27A\STAT1\STAT2* mRNA and miR-124 were normalized to *β-actin* and *U6*, respectively, which were used as internal controls. The ^△△^Ct method was used to determine the relative expression of these mRNAs. The primer sequences used for the targeted gene mRNA detection are listed in Supplementary Table 5. The primers for miR-124 and *U6* were purchased from RiboBio Co., Ltd. (Guangzhou, China).

### Western blotting analysis

Cells were harvested and lysed using RIPA lysis buffer (Cell Signaling Technology, Danvers, MA, USA) containing 1% protein protease and phosphatase inhibitor cocktail (Sigma-Aldrich, St. Louis, MO, USA) and incubated for 30 min. Samples were obtained with centrifugation at 12,000 rpm at 4 °C for 15 min and then heated for 5 min at 95 °C. Western blotting assays were performed as previously described^38^. The catalog numbers and dilution of primary antibodies are listed in Supplementary Table 6. For the IFNα treatment assay, cells were treated with IFNα at different concentrations and harvested at the indicated times. All blots and gels were derived from the same experiment and were processed in parallel.

### Cell proliferation analysis and drug treatment

The Cell Counting Kit-8 was used for cell proliferation analysis (APExBIO, USA). *RAB27A* overexpression stable cells and cells transfected with target siRNA or the corresponding negative controls were seeded into 96-well plates grown for 24, 48, and 72 h. Cell viability was determined according to the kit manufacturer’s instructions. Clone formation assay was used to detect cell proliferation. Briefly, the cells were diluted using a complete culture medium, and 3 × 10^3^ cells were seeded into 60-mm plates. After 7–14 days incubation, depending on the cell growth rate, foci were stained using crystal violet (Beyotime) and counted. For the drug treatment assay, cells were plated into 96-well plates, and cerdulatinib (#S3566; Selleck Chemicals, Houston, TX, USA) was added to the culture medium. After 72 h treatment, cell viability was assessed.

### Transwell assays

For migration and invasion assays in vitro, 30,000 cells in 200 μL RPMI-1640 medium containing 1% FBS were seeded into the upper chamber of a transwell insert or onto a Matrigel-coated transwell insert (BD Science, Sparks, MD, USA). RPMI-1640 medium (800 μL) containing 10% FBS was placed in the lower chamber as a chemoattractant. After incubation in a humidified atmosphere containing 5% CO_2_ at 37 °C for 24 h, the non-migrated or non-invaded cells were gently washed with cold PBS twice and removed from the upper chamber using cotton swabs. Methanol was used for the fixation of the remaining cells that had migrated or invaded the lower chamber for 15 min and stained using crystal violet. The cells were photographed and counted in three randomly selected visual fields. For the inhibitor treatment assay, cerdulatinib was used to treat cells for 24 h in six-well plates before the transwell assay. The cells were photographed and counted.

### Flow cytometric analysis

For the cell cycle analysis, cells with *RAB27A* stably overexpressing or transfected with siRNA were washed using cold PBS and trypsinized. After centrifugation at 3000 rpm for 5 min, cells were washed using cold PBS, and cold 70% ethanol was used for cell fixation at −20 °C overnight. Then the ethanol was discarded, and cells were washed with cold PBS and incubated with propidium iodide (Beyotime) for 30 min at 37 °C. A FACS Caliber system (Beckman Coulter, Brea, CA, USA) was used to analyze the stained cells. The gating strategy for flow cytometry analysis in Supplementary Figs. 1c and 4b was provided in Supplementary Fig. 8.

### Immunofluorescence staining

A549, H226, and H1975 cells were trypsinized, plated into 24-well plates with slides on the bottom for 24 h culture, and washed using cold PBS. 4% paraformaldehyde was used for cell fixation for 30 min, and cells were permeabilized using 0.5% Triton X-100 for 15 min, then blocked using 3% BSA for 60 min at 20 °C. After blocking, a Rab27A mouse primary antibody was diluted to 1:200 with 3% BSA, then added to the plate and incubated overnight at 4 °C. After discarding the Rab27A primary antibody, the cells were washed using cold PBS and incubated with FITC-conjugated secondary antibody (Invitrogen) for 1 h at 20 °C. Cell nuclei were stained with Hoechst (Beyotime), and images were captured using a confocal microscope (Nikon Eclipse Ti, Tokyo, Japan).

### Isolation and characterization of exosomes

*RAB27A*-knockdown stable cells (80 million cells) were cultured in the exosome-free medium for 48 h. Then exosomes derived from cells were isolated from 50 mL conditioned medium by ultracentrifugation. Briefly, the harvested medium was centrifuged at 4 °C, 500 × *g* for 5 min to collect the culture supernatant, and then at 2000 × *g* for 20 min to remove the cell debris. The collected culture supernatant was filtered using a 0.22-μm filter unit to remove large particles, and the remaining supernatant was ultracentrifuged for 70 min at 120,000 × *g* by using a Type 70 Ti rotor (Beckman Coulter Optima L-XP) at 4 °C. To eliminate contaminating proteins, the resulting pellet (exosomes) was resuspended in sterile PBS and centrifuged at 120,000 × *g* for 70 min. RIPA lysis buffer was used for the lysis of exosomes, and the expression level of the exosome marker (CD9) was examined using Western blotting analysis. Isolated exosomes were also subjected to NanoSight tracking analysis (NTA) to measure the distribution of particle size and zeta potential of the exosomes and transmission electron microscopy (TEM) (JEM-1200EX, JEOL, Japan) to classify their morphology.

### Luciferase reporter assay

A 268 bp sequence of the *HSPA5* promoter containing a putative STAT1 binding sequence (ttcctggaat) or mutated target sequence (aacgtctctt) was synthesized and directly subcloned into the pGL3 basic vector (Promega, Madison, WI, USA). The constructed and pRL-TK plasmids were co-transfected into HEK293T cells for 48 h before the cells were harvested, and the luciferase activity was examined with a Dual Luminescence Assay Kit (Promega).

To analyze the regulation of miR-124 on *RAB27A*, a 235 bp fragment containing the predicted miR-124 binding sites from the *RAB27A* 3’-UTR was synthesized and subcloned into the psiCHECK-2 luciferase reporter (Promega) to generate psiCHECK-2- RAB27A-3’-UTR wild-type or psiCHECK-2- RAB27A-3’-UTR mutant plasmids. A549, H1299, and H226 cells were transfected with wild-type or mutant plasmids using Lipofectamine 2000 (Life Technologies) for 48 h. The luciferase activity was evaluated as previously described^39^.

### Immunoprecipitation and mass spectrometry

Immunoprecipitation was performed by incubating the samples with anti-Flag M2 Affinity gel (Sigma-Aldrich) at 4 °C for over 4 h. The beads were then washed with cold lysis buffer and eluted with urea (8 M) in ammonium bicarbonate. Rab27A-interacting proteins were identified using mass spectrometry. For the label-free proteome, 100 μg protein of *RAB27A* knockdown stable cells were digested with a trypsin/Lys-C mixture. An Orbitrap Fusion Lumos Tribrid Mass Spectrometer was used to analyze all these samples (Thermo Fisher Scientific).

### Cytokine secretion assay

The supernatant of *RAB27A*-overexpressing, *RAB27A*-knockdown, and corresponding negative control cells was collected after 24 h. For the exosome secretion inhibition experiment, the cells were cultured with RPMI-1640 medium containing 1% FBS for 8–12 h and then treated with 50 nM 5-(N, N-dimethyl)-amiloride DMA (APExBIO, C3505) to inhibit exosome production. The culture supernatant was collected after 24 h, and ELISA assay was performed to measure the levels of IFNα, IFNβ, and IL-12.

### Immunocytochemistry assays

Immunohistochemical (IHC) assays were performed as described previously^39^. Tissue microarrays of 180 NSCLC tissues and matched adjacent normal tissues (90 pairs of adenocarcinomas: HLugA180Su02; 90 pairs of squamous cell carcinoma: HLugS180Su01; Outdo Biotech, Shanghai, China) were used to evaluate Rab27A protein expression. Tissue microarrays (ZL-LugA961) were obtained from Zhuolibiotech (Shanghai, China) to detect Rab27A and HSPA5 protein levels.

### In vivo growth and metastasis assays

Female BALB/C athymic nude mice of 6–8 weeks old were purchased to establish the NSCLC xenograft model, A549/Sh-NC and A549/Sh-Rab27A stably overexpressing cells were suspended in 100 μL PBS and injected subcutaneously into the armpits of the nude mice. Tumor volume (*V*) was measured every 2–3 days with Vernier calipers and applying the measurements. After 33 d, the mice were euthanized, and xenograft tumors were dissected for subsequent experiments.

For the in vivo cerdulatinib treatment assay, A549/Vector or A549/OE-Rab27A cells were injected into the flank sides of the nude mice. After 14 days, A549/OE-Rab27A treated animals were divided into two groups randomly (six mice in each group): a castor oil control group and a cerdulatinib treatment group (35 mg/kg). Castor oil and cerdulatinib were orally administered to the mice 5 days per week for 4 weeks. For in vivo metastasis assays, a total of 1.5 × 10^6^ A549/Vector or A549/OE-Rab27A cells were suspended in 150 μL PBS per mouse and injected into the tail vein of mice. After 7 days, A549/OE-Rab27A cells were divided into two groups randomly (five mice in each group) for castor oil and cerdulatinib administration following the above method. 6 weeks later, all mice were euthanized using the spinal dislocation method, and tumors, lungs, and live tissues from each mouse were excised and fixed with Bouin’s solution for detecting metastatic nodules. Hematoxylin and eosin (H&E) staining was used to analyze metastatic tissues histologically. This study has complied with all relevant ethical regulations for animal testing and research. All animal experimental procedures received ethical approval from the Laboratory Animal Center of Soochow University (approval no. 201908A101).

### Statistical analysis

The difference in Rab27A and miR-124 expression between adjacent non-cancerous tissues (N) and NSCLC tissues (T) was analyzed using a paired two-tailed *t*-test. For in vitro experiments, differences between the two groups were assessed using an unpaired *t*-test (two-tailed). To determine the difference in cell growth, two-way ANOVA was used between the two groups. Differences were considered statistically significant at *P* < 0.05. GraphPad Prism 8.0.2 (GraphPad, San Diego, CA, USA) and the SPSS software (version 16.0; SPSS, Chicago, IL, USA) were used to perform statistical analyses.

### Reporting summary

Further information on research design is available in the Nature Research Reporting Summary linked to this article.

### Supplementary information

REPORTING SUMMARY

Supplementary Information

## Data Availability

The data analyzed in this study are available from the corresponding author upon reasonable request.
