# Supplementary material for: Targeting TYK2 alleviates Rab27A-induced malignant progression of non-small cell lung cancer via disrupting IFNα-TYK2-STAT-HSPA5 axis
Source: NPJ Precis Oncol. 2024 Mar 23;8:74. doi: 10.1038/s41698-024-00574-1 (PMC10960821; doi:10.1038/s41698-024-00574-1)
Supplement: Supplementary file 1 — REPORTING SUMMARY [file 41698_2024_574_MOESM1_ESM.pdf]

nature portfolio

Corresponding author(s): Jian-an Huang, Zeyi Liu

Last updated by author(s): Feb 24, 2024

Reporting Summary

Nature Portfolio wishes to improve the reproducibility of the work that we publish. This form provides structure for consistency and transparency in reporting. For further information on Nature Portfolio policies, see our Editorial Policies and the Editorial Policy Checklist.

Statistics

For all statistical analyses, confirm that the following items are present in the figure legend, table legend, main text, or Methods section.

n/a	Confirmed	
☐	☒	The exact sample size (*n*) for each experimental group/condition, given as a discrete number and unit of measurement
☐	☒	A statement on whether measurements were taken from distinct samples or whether the same sample was measured repeatedly
☐	☒	The statistical test(s) used AND whether they are one- or two-sided<br>*Only common tests should be described solely by name; describe more complex techniques in the Methods section.*
☒	☐	A description of all covariates tested
☒	☐	A description of any assumptions or corrections, such as tests of normality and adjustment for multiple comparisons
☒	☐	A full description of the statistical parameters including central tendency (e.g. means) or other basic estimates (e.g. regression coefficient) AND variation (e.g. standard deviation) or associated estimates of uncertainty (e.g. confidence intervals)
☐	☒	For null hypothesis testing, the test statistic (e.g. *F*, *t*, *r*) with confidence intervals, effect sizes, degrees of freedom and *P* value noted<br>*Give P values as exact values whenever suitable.*
☒	☐	For Bayesian analysis, information on the choice of priors and Markov chain Monte Carlo settings
☒	☐	For hierarchical and complex designs, identification of the appropriate level for tests and full reporting of outcomes
☒	☐	Estimates of effect sizes (e.g. Cohen's *d*, Pearson's *r*), indicating how they were calculated

Our web collection on statistics for biologists contains articles on many of the points above.

Software and code

Policy information about availability of computer code

Data collection	The expression data of RAB27A was downloaded from the GEO database (http://www.ncbi.nih.gov/geo).
Data analysis	No software was used.

For manuscripts utilizing custom algorithms or software that are central to the research but not yet described in published literature, software must be made available to editors and reviewers. We strongly encourage code deposition in a community repository (e.g. GitHub). See the Nature Portfolio guidelines for submitting code & software for further information.

Data

Policy information about availability of data

All manuscripts must include a data availability statement. This statement should provide the following information, where applicable:

- Accession codes, unique identifiers, or web links for publicly available datasets
- A description of any restrictions on data availability
- For clinical datasets or third party data, please ensure that the statement adheres to our policy

We have no data that require deposition in a public database.

Research involving human participants, their data, or biological material

Policy information about studies with human participants or human data. See also policy information about sex, gender (identity/presentation), and sexual orientation and race, ethnicity and racism.

Reporting on sex and gender	This study had divided the patients into Male and Female. The expression of RAB27A was correlated with gender according to this study.
Reporting on race, ethnicity, or other socially relevant groupings	N/A
Population characteristics	N/A
Recruitment	Paired NSCLC tissue and adjacent noncancerous lung tissue samples were collected with informed consent from patients at the First Affiliated Hospital of Soochow University between 2015 and 2018.
Ethics oversight	All research protocols were approved by the Ethics Committee of the First Affiliated Hospital of Soochow University (approval no. 2018-255) and conducted in accordance with all relevant ethical regulations including the Declaration of Helsinki.

Note that full information on the approval of the study protocol must also be provided in the manuscript.

Field-specific reporting

Please select the one below that is the best fit for your research. If you are not sure, read the appropriate sections before making your selection.

☒ Life sciences ☐ Behavioural & social sciences ☐ Ecological, evolutionary & environmental sciences

For a reference copy of the document with all sections, see nature.com/documents/nr-reporting-summary-flat.pdf

Life sciences study design

All studies must disclose on these points even when the disclosure is negative.

Sample size	Paired NSCLC tissue and adjacent noncancerous lung tissue samples (115 of each) were collected to detect RAB27A mRNA expression by QRT-PCR. 180 NSCLC tissues and matched adjacent normal tissues were used to evaluate Rab27A protein expression by Immunocytochemistry assay.
Data exclusions	6 NSCLC tissues and matched adjacent normal tissues involved in Immunocytochemistry assay were excluded. Because these samples were damaged during the experiment and not used to evaluate Rab27A protein expression exactly.
Replication	All attempts at replication were successful.
Randomization	All tissue samples were allocated as paired tumor and normal from the same patient.
Blinding	Blinding was not relevant to our study because paired tumor and normal tissue were got from the same patient.

Reporting for specific materials, systems and methods

We require information from authors about some types of materials, experimental systems and methods used in many studies. Here, indicate whether each material, system or method listed is relevant to your study. If you are not sure if a list item applies to your research, read the appropriate section before selecting a response.

Materials & experimental systems

n/a	Involved in the study	
☐	☒	Antibodies
☐	☒	Eukaryotic cell lines
☒	☐	Palaeontology and archaeology
☐	☒	Animals and other organisms
☒	☐	Clinical data
☒	☐	Dual use research of concern
☒	☐	Plants

Methods

n/a	Involved in the study	
☒	☐	ChIP-seq
☐	☒	Flow cytometry
☒	☐	MRI-based neuroimaging

Antibodies

Antibodies used	Rab27A (Cell Signaling Technology, #69295S; Abcam, #ab55667), CD9 (Cell Signaling Technology, #13403S), CD81 (Santa Cruz Biotechnology, #sc-166029), TSG101 (Proteintech, #67381-1-Ig), GM130 (Proteintech, #11308-1-AP), Calnexin (Proteintech, #10427-2-AP), MMP2 (Proteintech, #10373-2-AP), MMP9 (Cell Signaling Technology, #13667S), N-cadherin (Proteintech, #22018-1-AP), Snail (Cell Signaling Technology, #3895S), Cyclin D1 (Cell Signaling Technology, #55506S), Cyclin A2 (Proteintech, #18202-1-AP), GSK3β (Cell Signaling Technology, #12456T), p-GSK3β (Ser9) (Cell Signaling Technology, #5558T), TYK2 (Cell Signaling Technology, #14193S), p-TYK2 (Tyr 1054/1055) (Cell Signaling Technology, #68790S), HSPA5(Proteintech,#66574-1-Ig), PCNA (Proteintech, #60097-1-Ig), β-actin (Proteintech, #66009-1-Ig).
Validation	All the primary antibodies used in these studies were validated by the manufacturers. The catalog numbers and dilution of antibodies have been listed in Supplementary Table 6.

Eukaryotic cell lines

Policy information about cell lines and Sex and Gender in Research

Cell line source(s)	All cell lines were purchased from Procell Life Science & Technology Co., Ltd (Wuhan, China).
Authentication	All cell lines were authenticated by STR DNA profiling analysis.
Mycoplasma contamination	All cell lines were detected to be free of mycoplasma contamination.
Commonly misidentified lines (See ICLAC register)	N/A

Animals and other research organisms

Policy information about studies involving animals; ARRIVE guidelines recommended for reporting animal research, and Sex and Gender in Research

Laboratory animals	BALB/c nude mice (6-8 weeks old; Female) were used in this study.
Wild animals	N/A
Reporting on sex	N/A
Field-collected samples	N/A
Ethics oversight	All animal experimental procedures recevied ethical approval from the Laboratory Animal Center of Soochow University (approval no. 201908A101).

Note that full information on the approval of the study protocol must also be provided in the manuscript.

Plants

Seed stocks	Our study does not relate to plants.
Novel plant genotypes	Our study does not relate to plants.
Authentication	Our study does not relate to plants.

Flow Cytometry

Plots

Confirm that:

☒ The axis labels state the marker and fluorochrome used (e.g. CD4-FITC).

☒ The axis scales are clearly visible. Include numbers along axes only for bottom left plot of group (a 'group' is an analysis of identical markers).

☒ All plots are contour plots with outliers or pseudocolor plots.

☒ A numerical value for number of cells or percentage (with statistics) is provided.

Methodology

Sample preparation	For the cell cycle analysis, cells with RAB27A stably overexpressing or transfected with siRNA were washed and trypsinized. After centrifugation at 3000 rpm for 5 min, cells were washed and cold 70% ethanol was used to cell fixation at -20°C overnight. Then the ethanol was discarded and cells were washed and incubated with propidium iodide (Beyotime) for 30 min at 37°C.
Instrument	Beckman Coulter FC500
Software	FlowJO X Software
Cell population abundance	Abundance of Propidium-PE positive cell popolation
Gating strategy	Based on isotype group

☒ Tick this box to confirm that a figure exemplifying the gating strategy is provided in the Supplementary Information.
