# Supplementary material for: Targeting TYK2 alleviates Rab27A-induced malignant progression of non-small cell lung cancer via disrupting IFNα-TYK2-STAT-HSPA5 axis
Source: NPJ Precis Oncol. 2024 Mar 23;8:74. doi: 10.1038/s41698-024-00574-1 (PMC10960821; doi:10.1038/s41698-024-00574-1)
Supplement: Supplementary file 2 — Supplementary Information [file 41698_2024_574_MOESM2_ESM.pdf]

Supplementary Figures

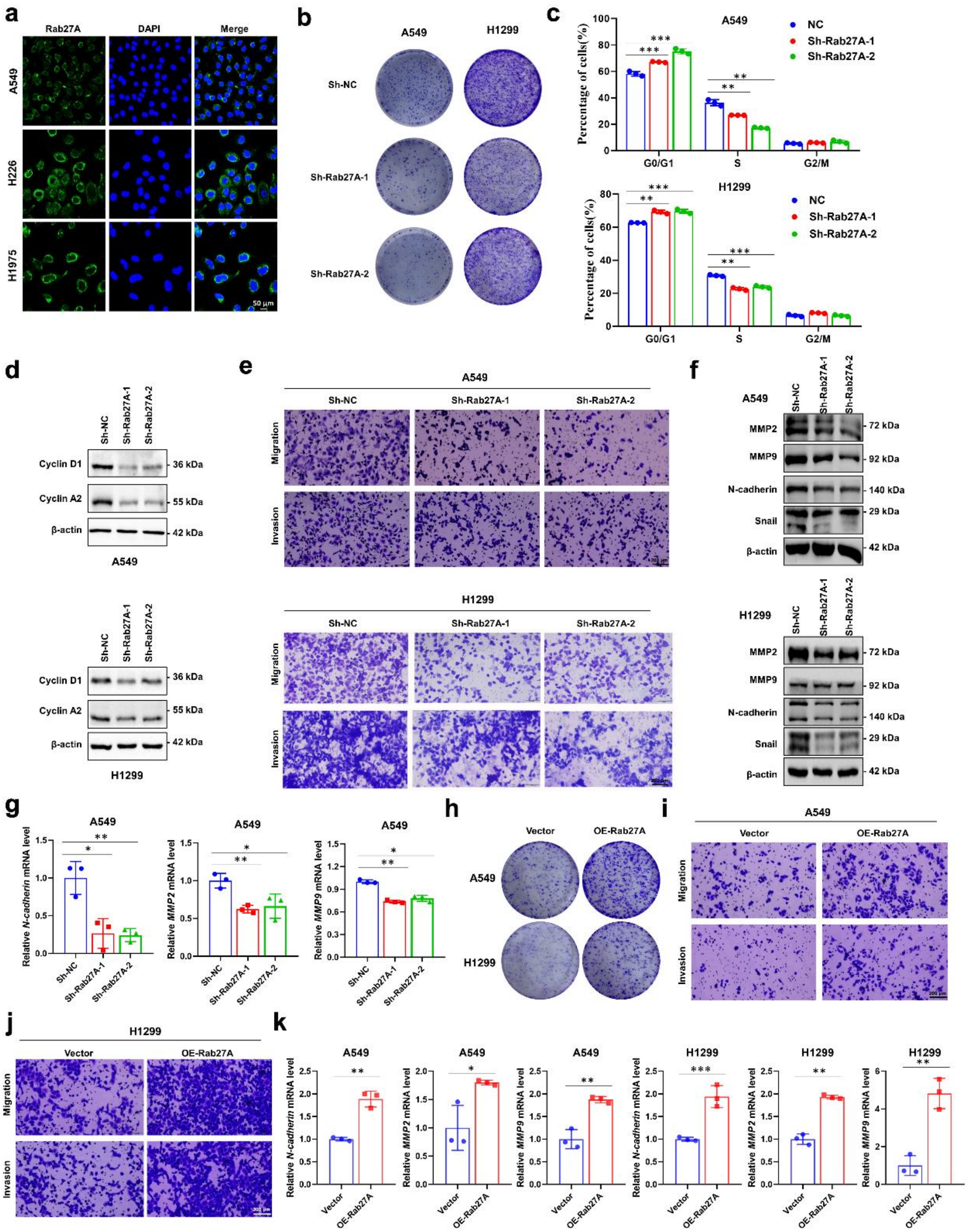

Supplementary Figure 1 a. Immunofluorescence assay showed that Rab27A was mainly localized in the cytoplasm of A549, H226 and H1975 cells (Scale bar, 50 μm). **b.** Representative images of the results of clonogenic analysis of cell proliferation in A549 and H1299 cells infected with the indicated shRNAs. **c.** Flow cytometric analysis of A549 and H1299 cells infected with the indicated shRNAs. Cells were harvested and stained with PI. The percentage of cells in each cell cycle phase is shown in the inset of each panel. **d.** Cell lysates from A549 and H1299 cells infected with the indicated shRNAs were

subjected to WB analysis to detect the expression of Cyclin D1 and Cyclin A2. **e.** Representative images of the results of Transwell analysis of cell migration and invasion ability in A549 and H1299 cells infected with the indicated shRNAs (Scale bar, 200 μm). **f.** Cell lysates from A549 and H1299 cells infected with the indicated shRNAs were subjected to Western blot analysis to detect the protein level of EMT markers (MMP2, MMP9, N-Cadherin and Snail). **g.** qRT-PCR analysis of EMT markers (N-Cadherin, MMP2 and MMP9) in A549 and H1299 cells infected with the indicated shRNAs. **h** Representative images of the results of clonogenic analysis of cell proliferation in A549 and H1299 cells infected with the indicated lentiviral vectors. **i-j.** Representative images of the results of Transwell analysis of cell migration and invasion ability in A549 and H1299 cells infected with the indicated lentiviral vectors (Scale bar, 200 μm). **k.** qRT-PCR analysis of EMT markers (N-Cadherin, MMP2 and MMP9) in A549 and H1299 cells infected with the indicated lentiviral vectors. **P* < 0.05; ***P* < 0.01; ****P* < 0.001

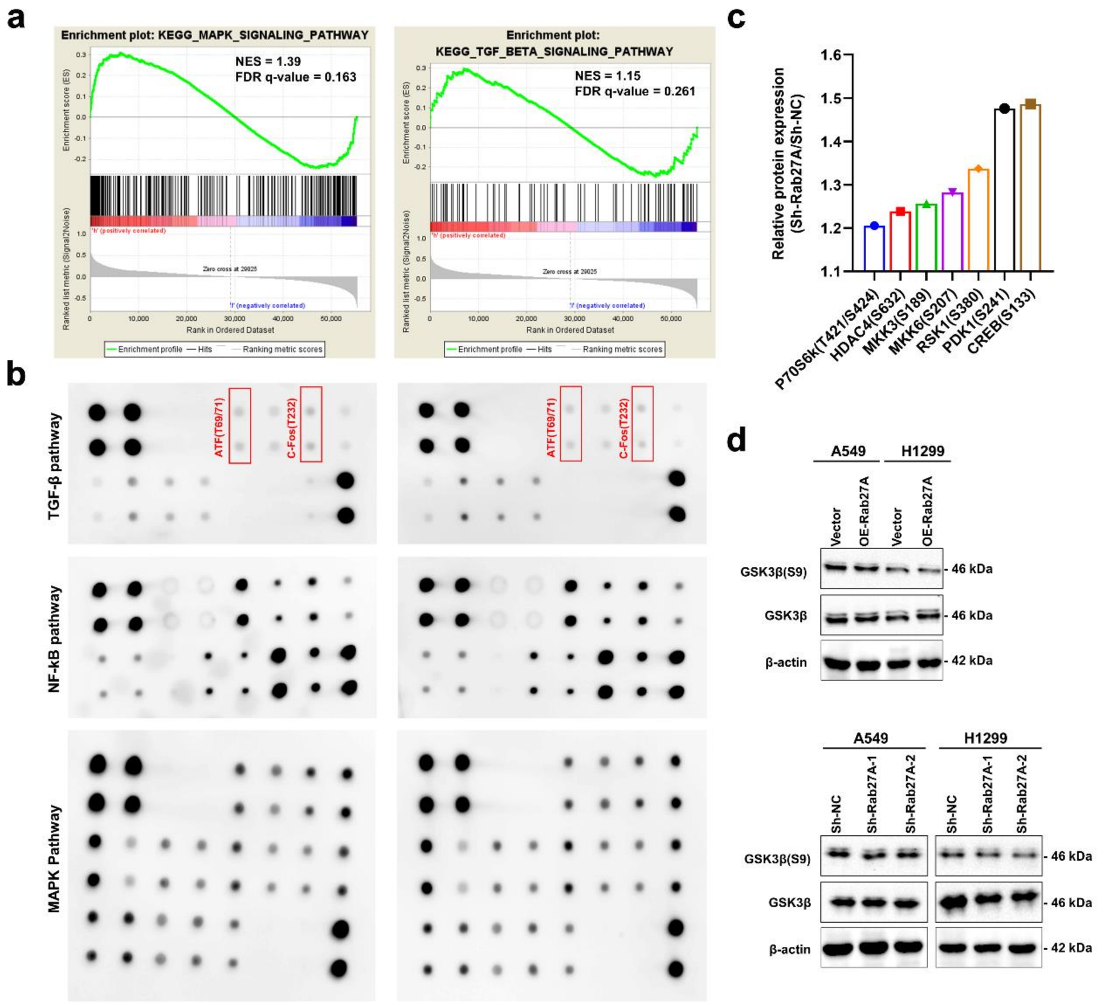

Supplementary Figure 2 a. GSEA analysis based on TCGA data suggested that the high-level expression of Rab27A is related to the activation of the MAPK signaling pathway and TGF-β signaling pathway. **b.** Plot map of Phosphorylation Pathway Profiling Array indicated the phosphorylation activation of TGF-β signaling pathway, NF-κB signaling pathway, and MAPK signaling pathway. **c.** Relative protein expression level of Rab27A-knockdown cells

and negative control cells based on Phosphorylation Pathway Profiling Array. **d.** Cell lysates from A549 and H1299 cells infected with the indicated lentiviral vectors and shRNAs were subjected to WB analysis to detect the expression of GSK3β.

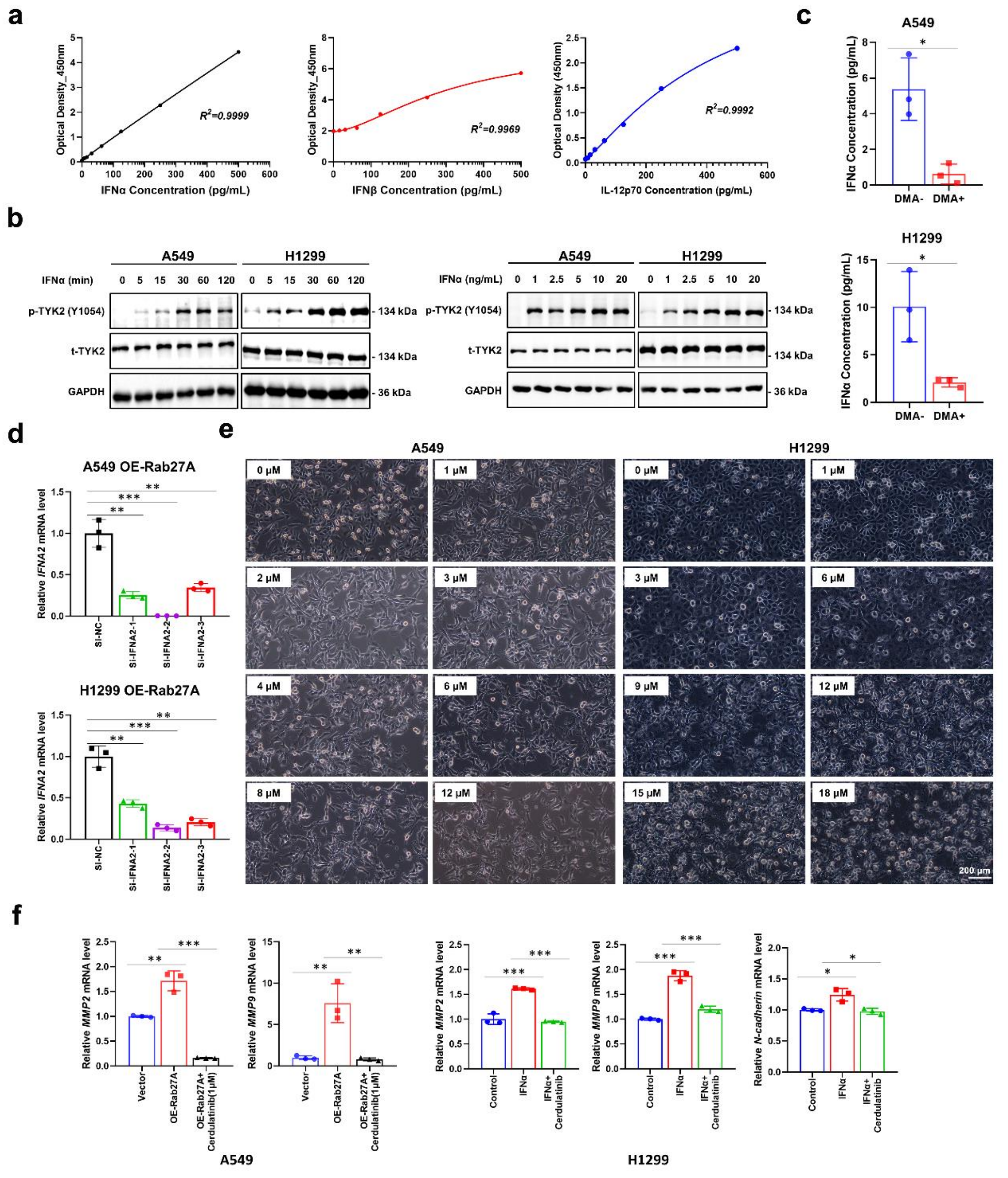

Supplementary Figure 3 a. Standard curve of ELISA to detect the concentration of IFNα, IFNβ, and IL-12p70 in the supernatant of A549 and H1299 cells. **b.** Cell lysates from A549 and H1299 cells treated with IFNα for the indicated time and concentration were subjected to Western blot analysis to detect the phosphorylation level of TYK2 (Y1054). **c.** Exosome secretion inhibitor DMA treatment significantly reduced the IFNα concentration in the cell supernatant of A549 and H1299 cells. **d.** IFNA2 mRNA and protein levels in IFNA2-knockdown NSCLC cells and negative control cells. **e.** After treatment with the indicated concentrations of Cerdulatinib, the growth and morphology of A549 and H1299 cells were

significantly affected (Scale bar, 200 μm). **f.** qRT-PCR analysis of EMT markers (N-Cadherin, MMP2, and MMP9) in A549 and H1299 cells. *P < 0.05; **P < 0.01; ***P < 0.001

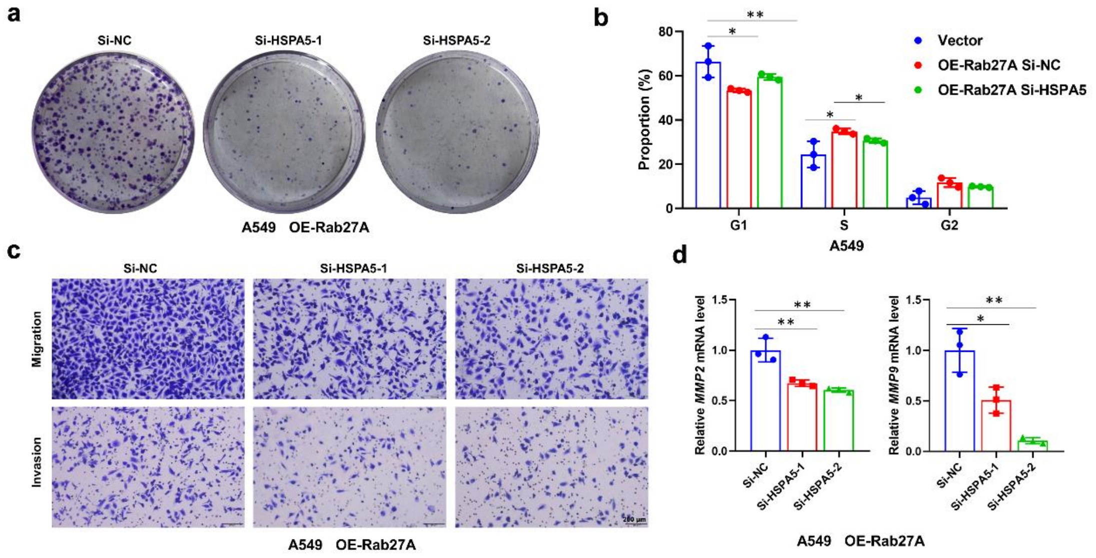

Supplementary Figure 4 a. Representative image of the results of clonogenic analysis of cell proliferation in A549 cells overexpressing Rab27A with HSPA5-knockdown. **b.** Flow cytometric analysis of A549 cells overexpressing Rab27A with HSPA5-knockdown. Cells were harvested and stained with PI. The percentage of cells in each cell cycle phase is shown in the inset of each panel. **c.** Representative images of the results of Transwell analysis of cell migration and invasion ability in A549 cells overexpressing Rab27A with HSPA5-knockdown (Scale bar, 200 μm). **d.** qRT-PCR analysis of MMP2, and MMP9 in A549 cells overexpressing Rab27A with HSPA5-silencing. *P < 0.05; **P < 0.01; ***P < 0.001

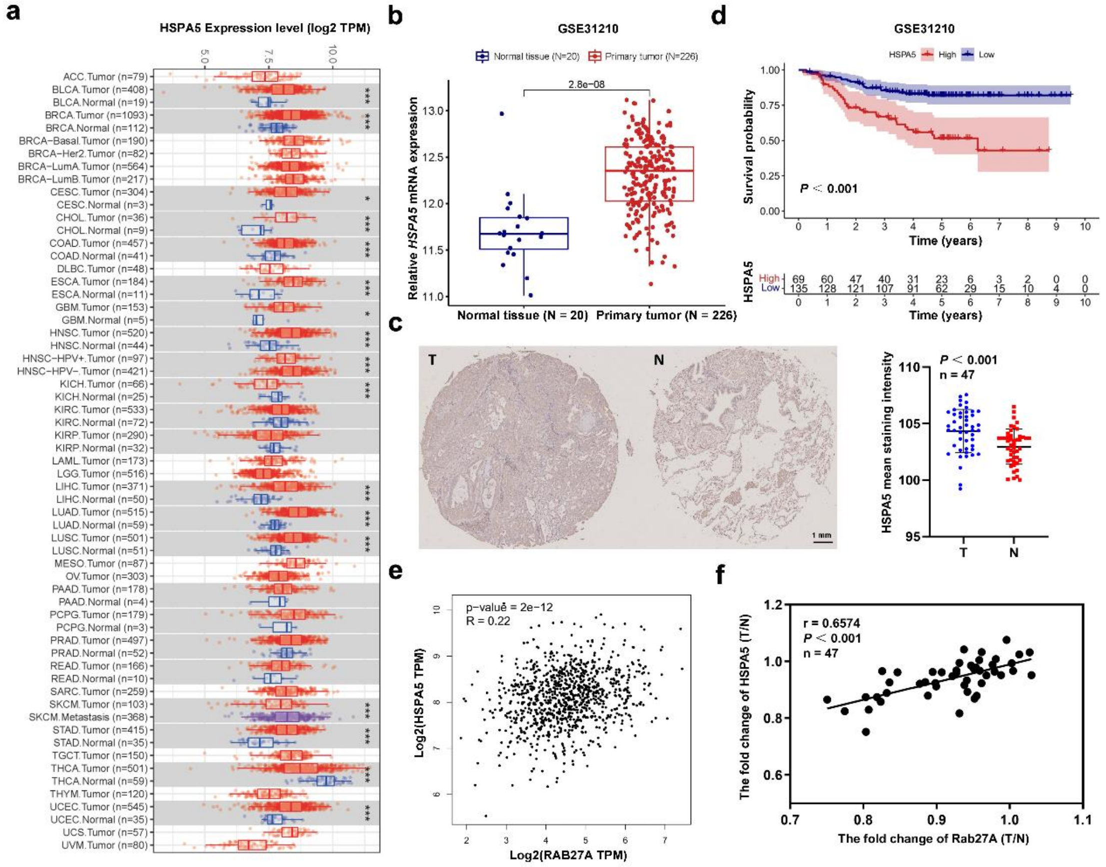

Supplementary Figure 5 a. HSPA5 mRNA expression in pan-cancer (http://timer.cistrome.org/). **b.** The expression data of HSPA5 was downloaded from the GEO database (http://www.ncbi.nih.gov/geo), and was analyzed to compared the expression differences between NSCLC tissues (N=226) and paired normal lung tissues (N=20) (Microarray ID: GSE31210). **c.** Formalin-fixed and paraffin-embedded human NSCLC tissues and paired normal lung tissues were subjected to IHC to visualize the expression of HSPA5 protein. The HSPA5 mean staining intensity was compared between NSCLC tissues (T) and paired normal lung tissues (N) (Scale bar, 1 mm). **d.** Effect of the HSPA5 expression level on the overall survival of NSCLC patients. The expression data and prognostic data were downloaded from the GEO database (Microarray ID: GSE31210). **e.** The correlations between two genes (HSPA5 and RAB27A): The expression correlation of two genes was analyzed with Spearman (http://gepia.cancer-pku.cn/). **f.** Formalin-fixed and paraffin-embedded NSCLC tissues and paired normal lung tissues were performed IHC analyses of the HSPA5 and Rab27A protein. Correlation analysis was performed.

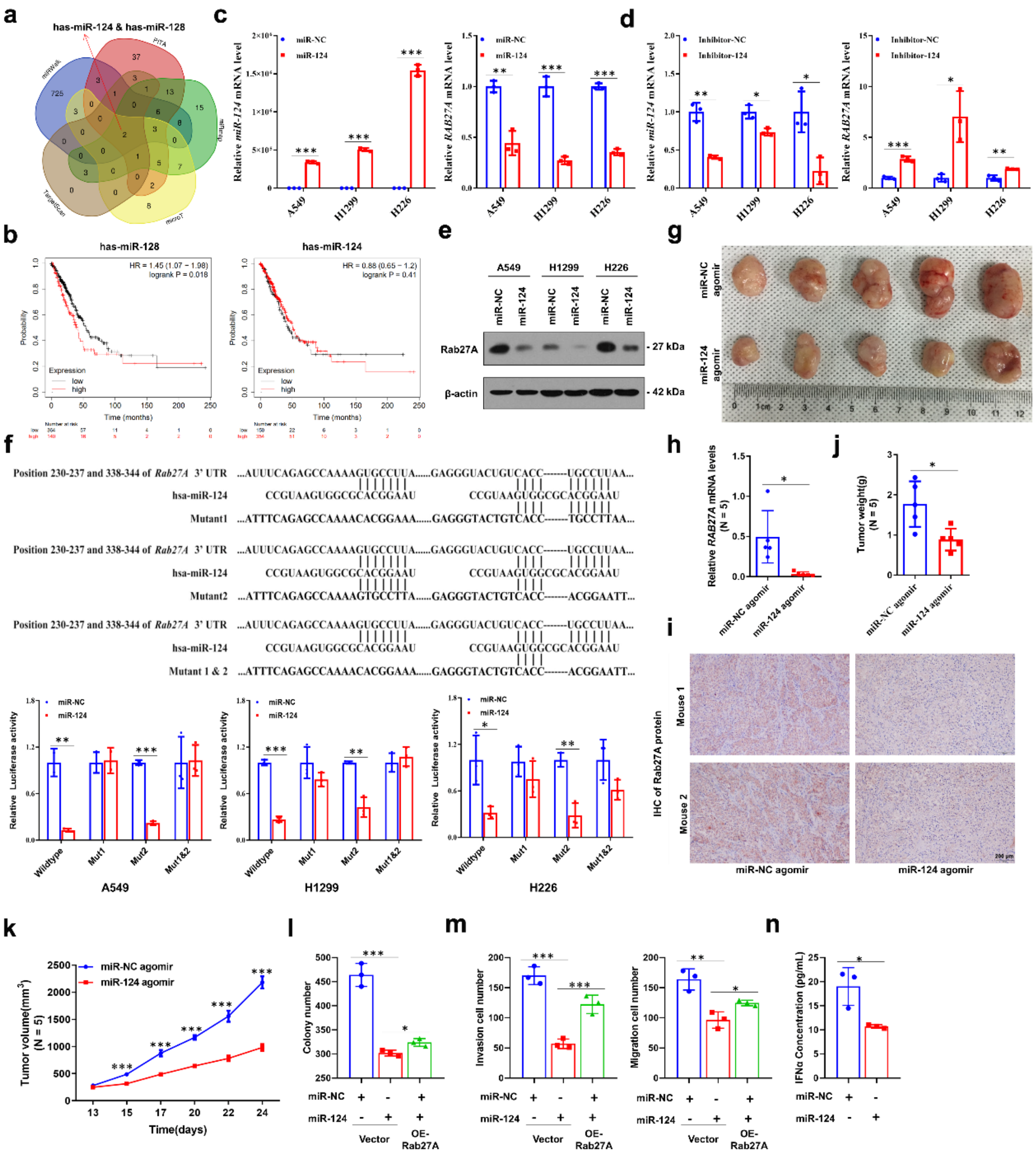

Supplementary Figure 6 a. miR-124 is predicted to combine with the 3'-UTR of Rab27A mRNA using five public databases (miRWalk, PITA, miRmap, microT, and Targetscan). **b.** Kaplan-Meier survival curves (http://kmplot.com/analysis/) of NSCLC patients (N=404) with high or low expression levels of miR-124 and miR-128. **c.** The expression level of Rab27A mRNA was inhibited in A549, H1299 and H226 cells transfected with miR-124. **d.** The expression level of Rab27A mRNA and protein was increased in A549, H1299 and H226 cells transfected with miR-124 inhibitor. **e.** The expression level of Rab27A protein was inhibited in A549, H1299 and H226 cells transfected with miR-124. **f.** Dual luciferase reporter (DLR) assay confirmed that miR-124 could combine with the 3'-UTR of Rab27A mRNA. **g.** A549 cells transfected with miR-124 agomir were inoculated into BALB/C athymic mice. At the experimental endpoint, tumors were dissected and photographed as shown. **h-i.** qRT-PCR and IHC to verify the mRNA and protein level of RAB27A in dissected tumors (Scale bar, 200 μm). **j.** Each tumor formed was weighted. **k.** Tumor growth curves in mice (n=5 in each group). **l-m.** The inhibiting effect of clonogenic, migration and invasion

mediated by miR-124 mimics could be reversed by Rab27A-overexpression. **n.** IFNα concentration decreased in H1299 RAB27A-overexpressing cells after transfection with miR-124 mimics. *$P < 0.05$; **$P < 0.01$; ***$P < 0.001$

Supplementary Figure 7 Uncropped scans were provided.

Figure 2a

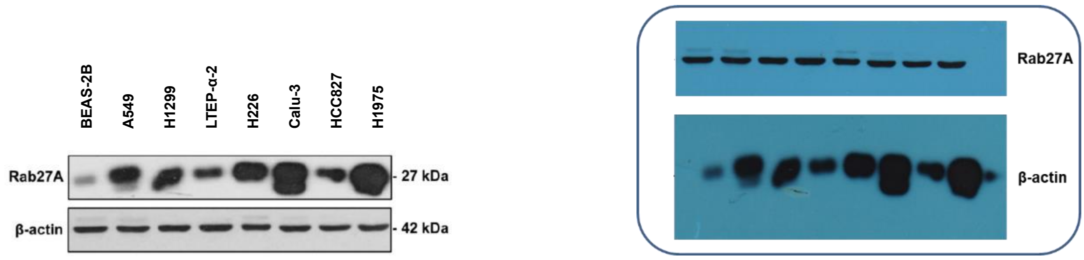

Figure 2b

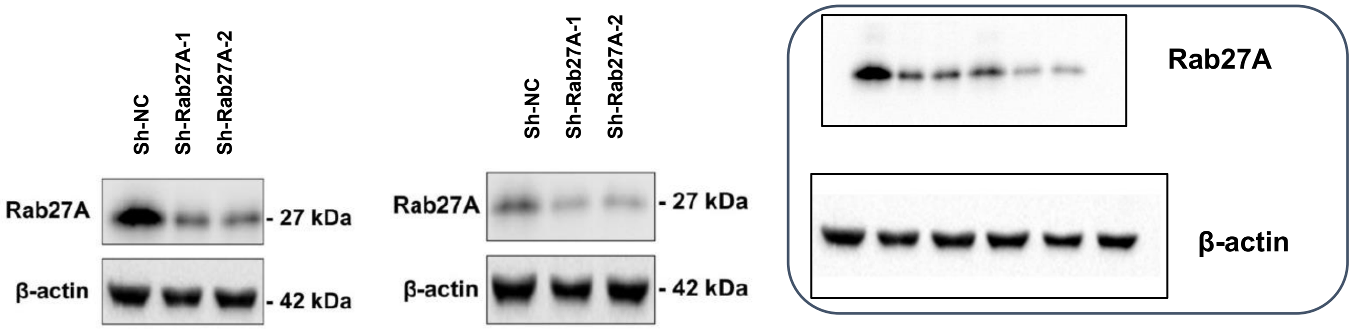

Figure 2c

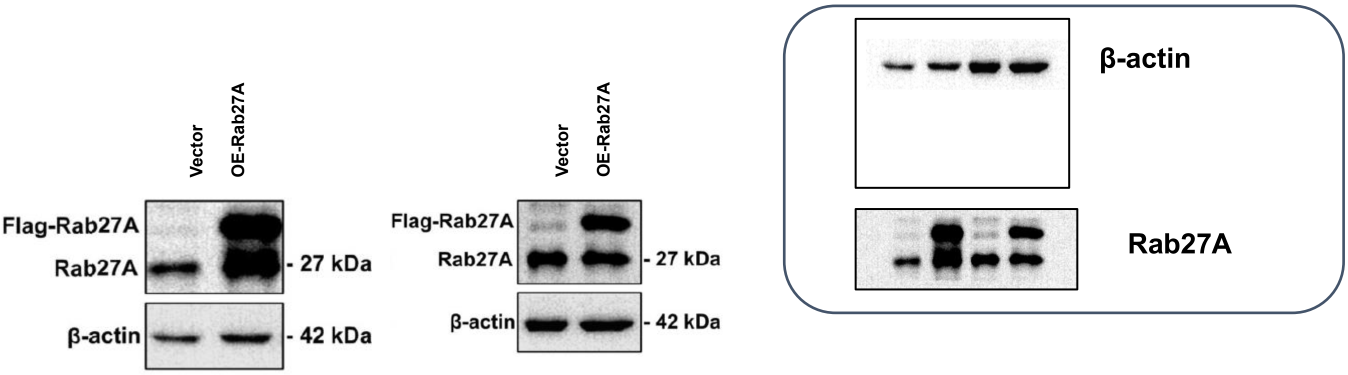

Figure 2f

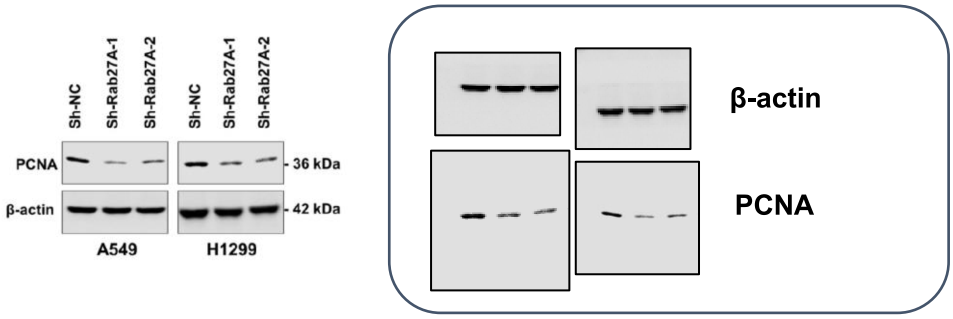

Figure 2k

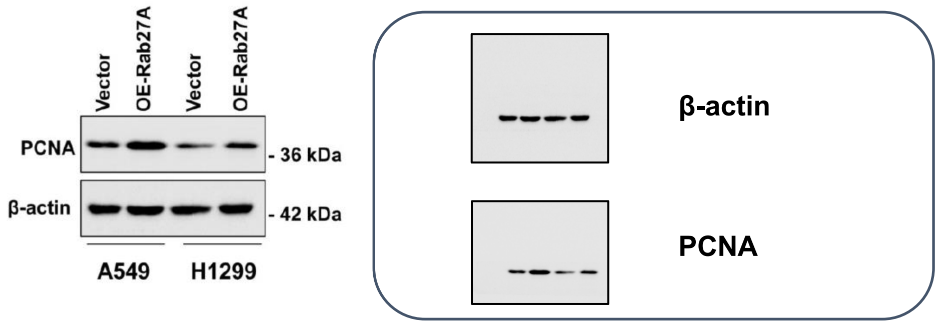

Figure 3d

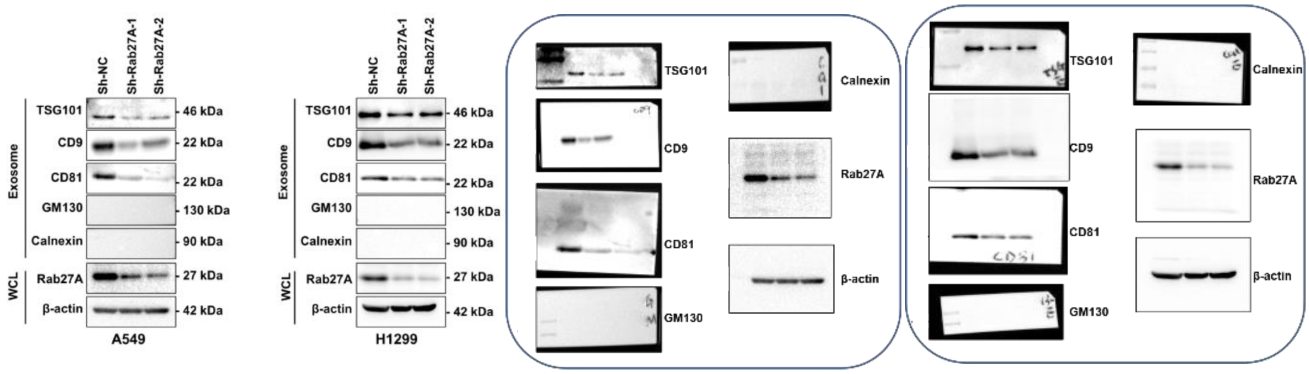

Figure 4e

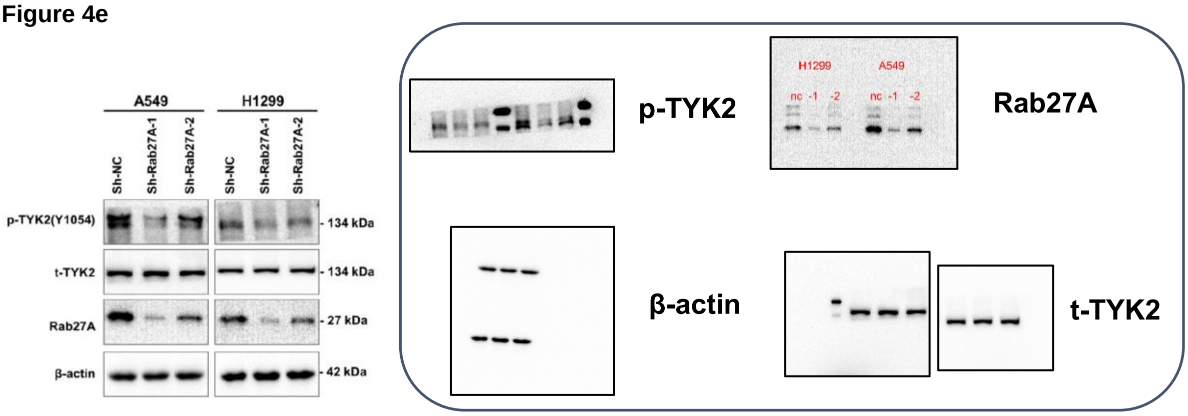

Figure 4g

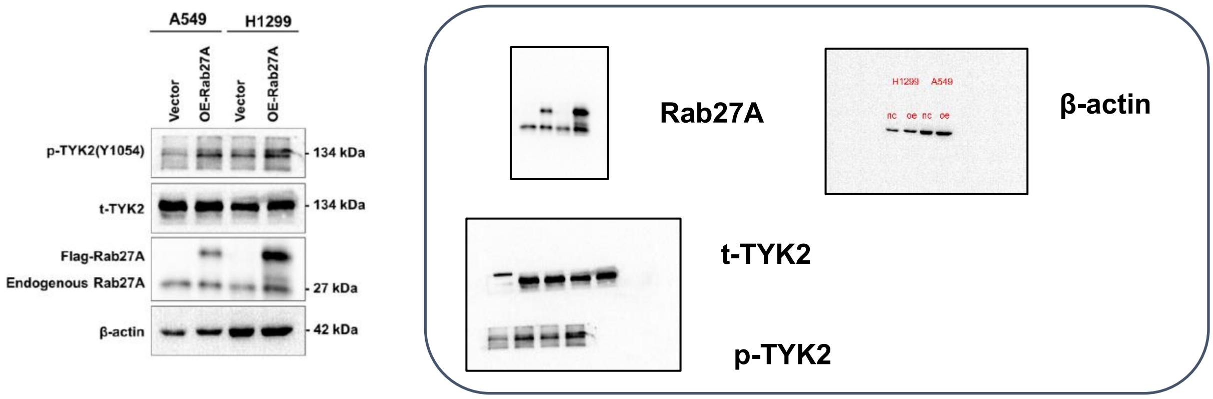

Figure 4i

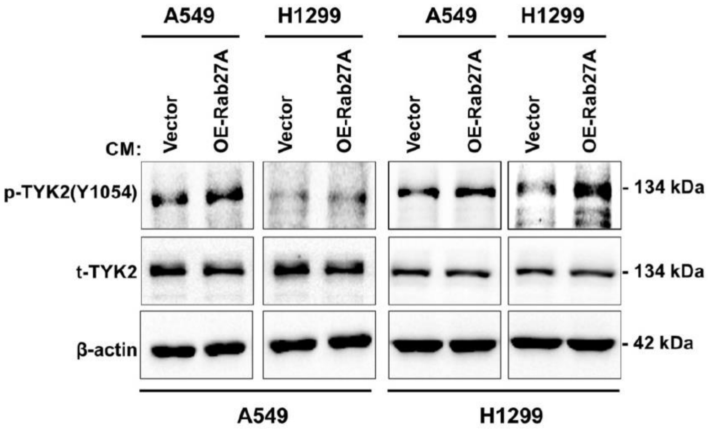

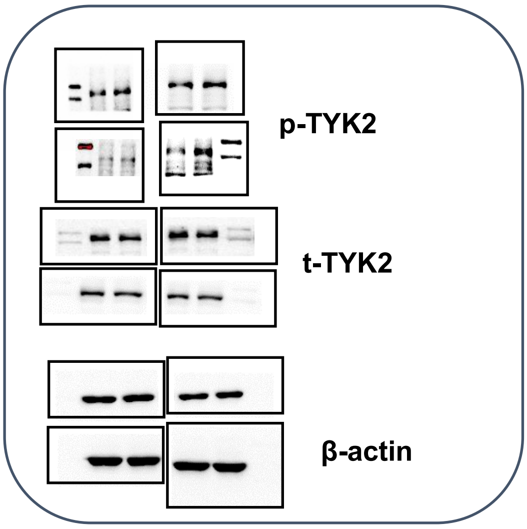

Figure 5d

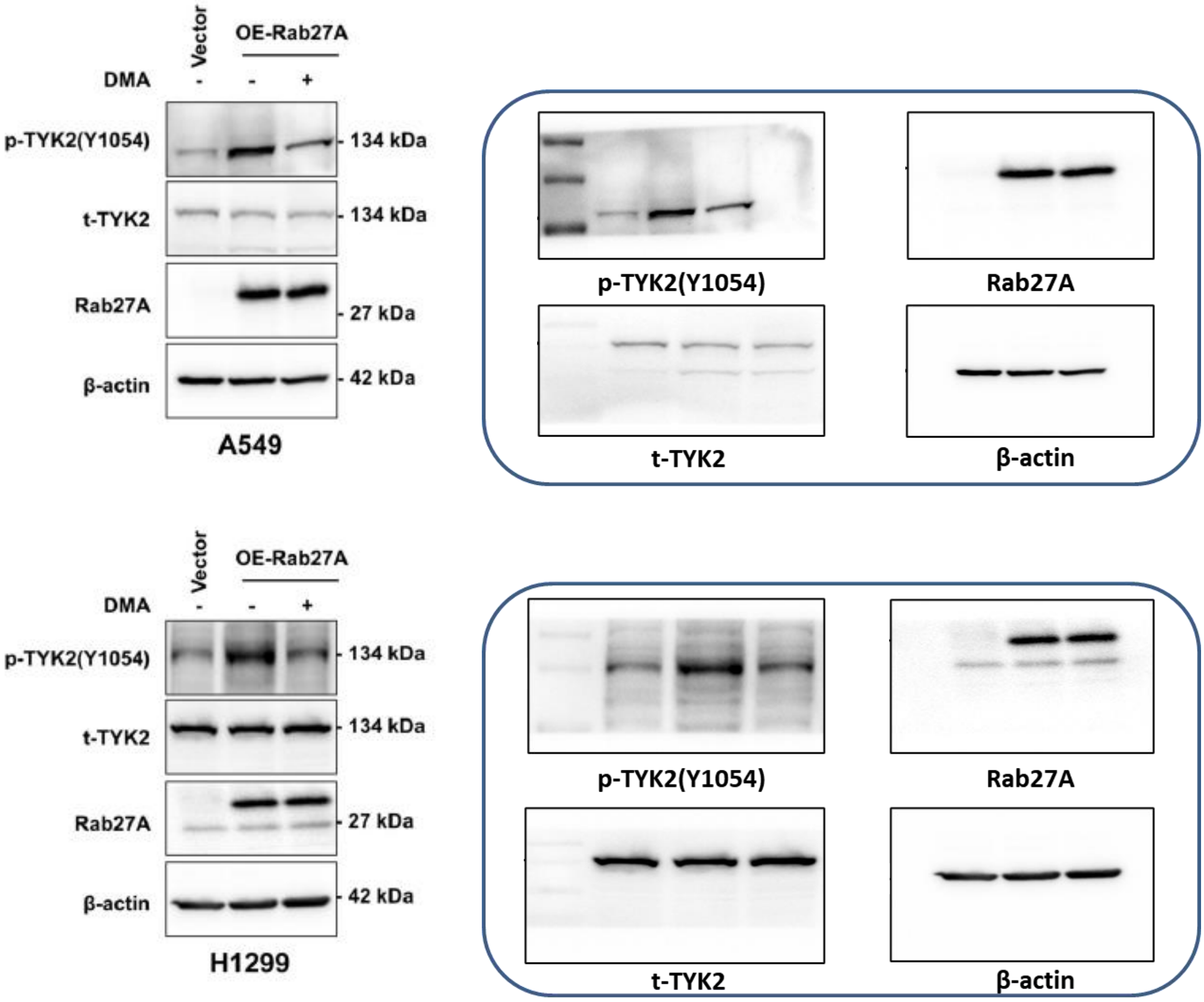

Figure 5f

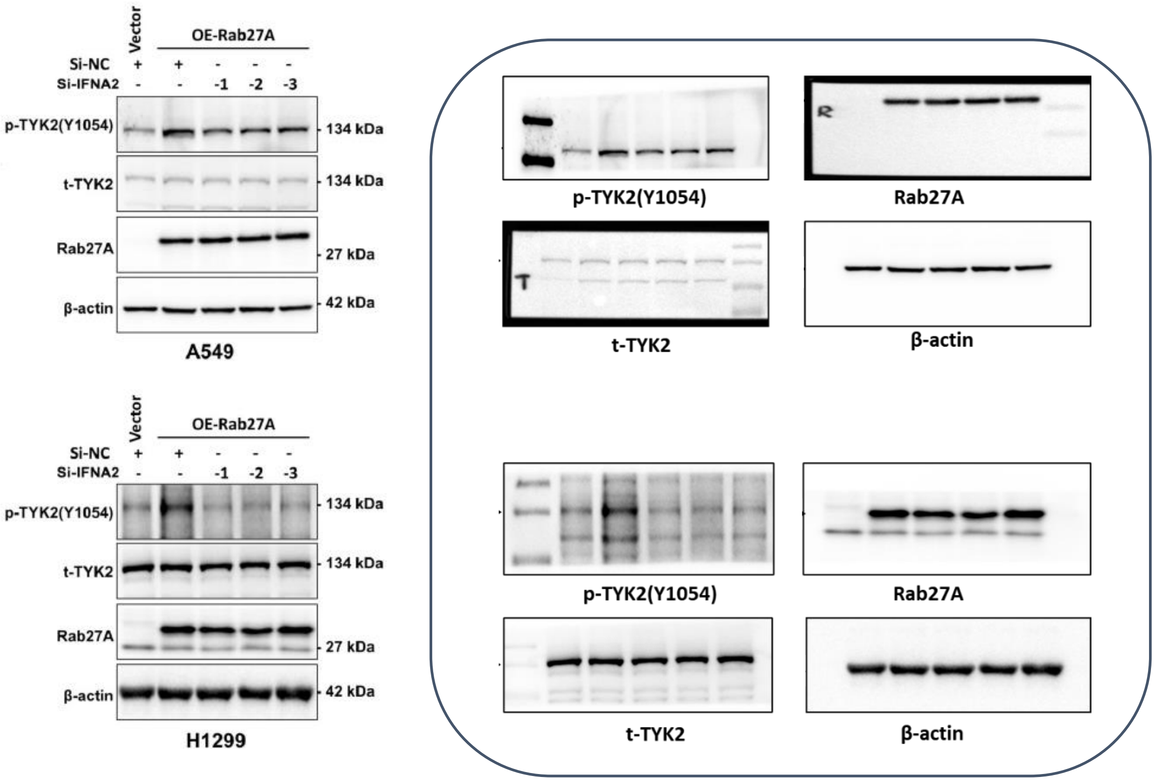

Figure 6b

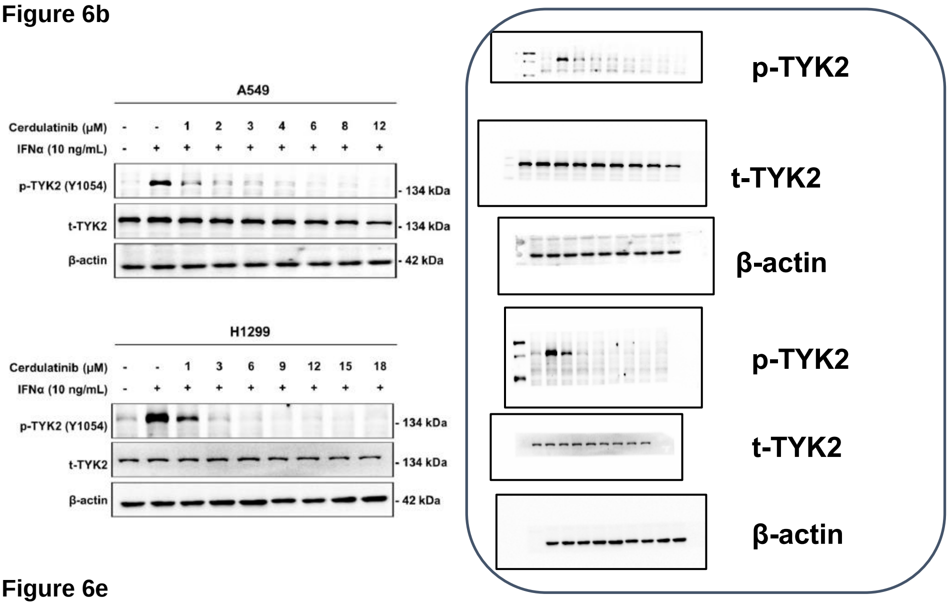

Figure 6e

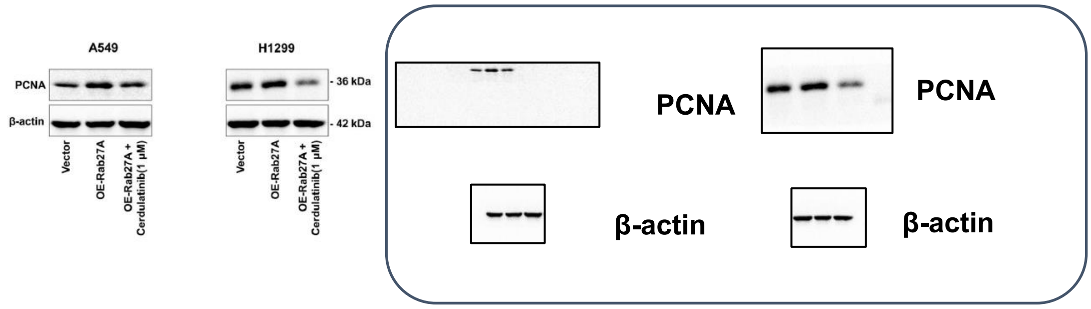

Figure 7d

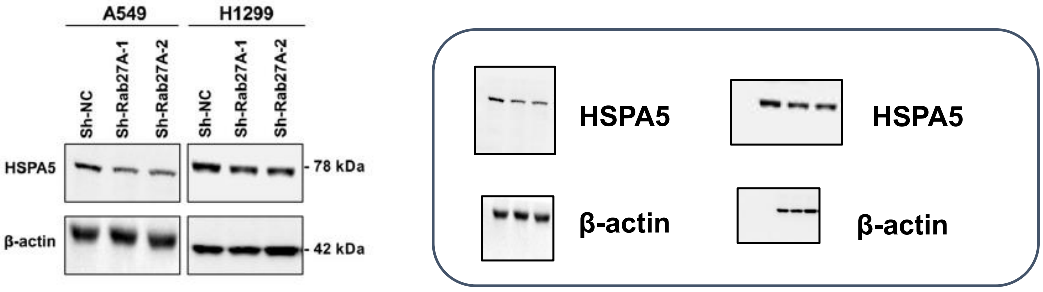

Figure 7e

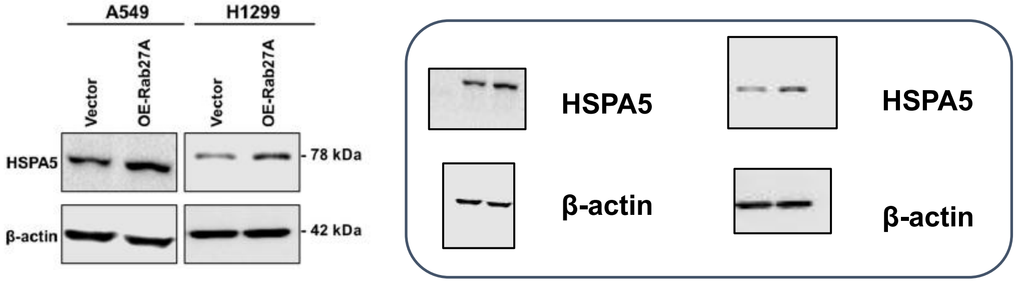

Figure 7i

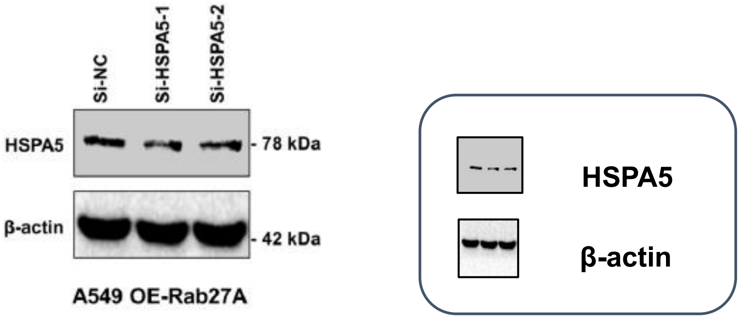

Figure 8b

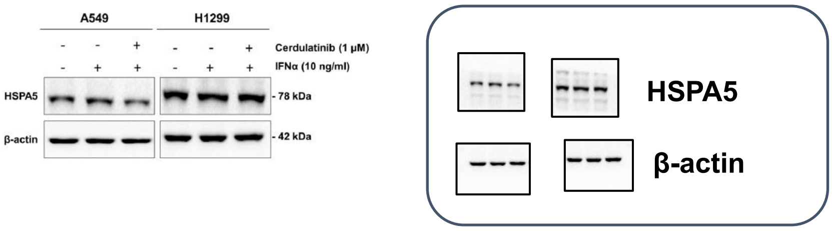

Supplementary Figure 1d

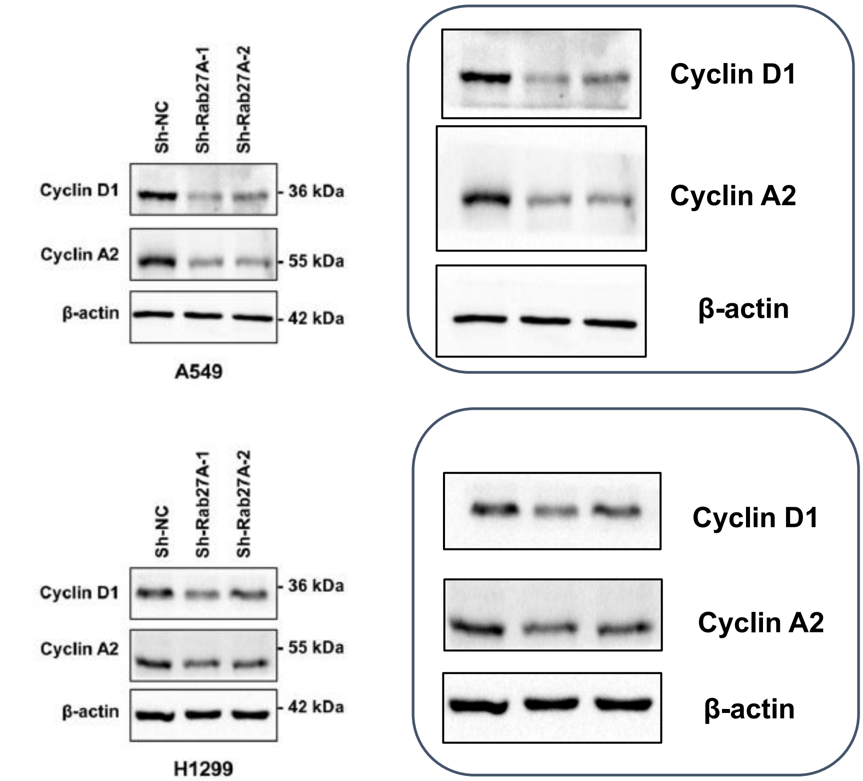

Supplementary Figure 1f

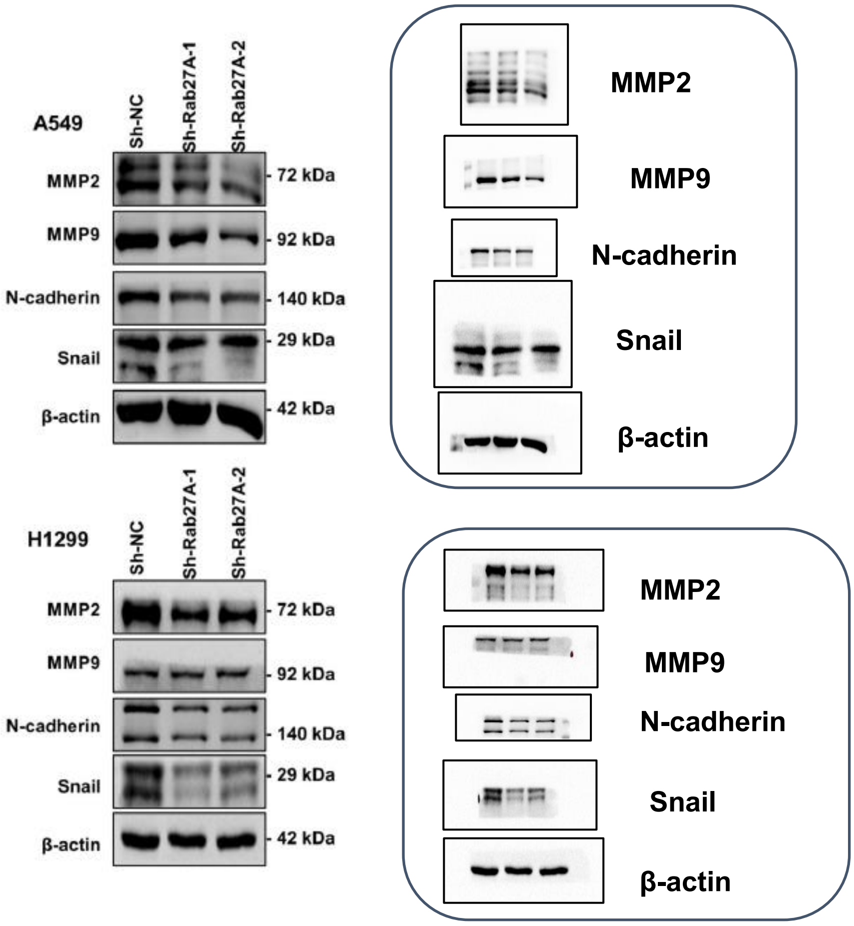

Supplementary Figure 2d

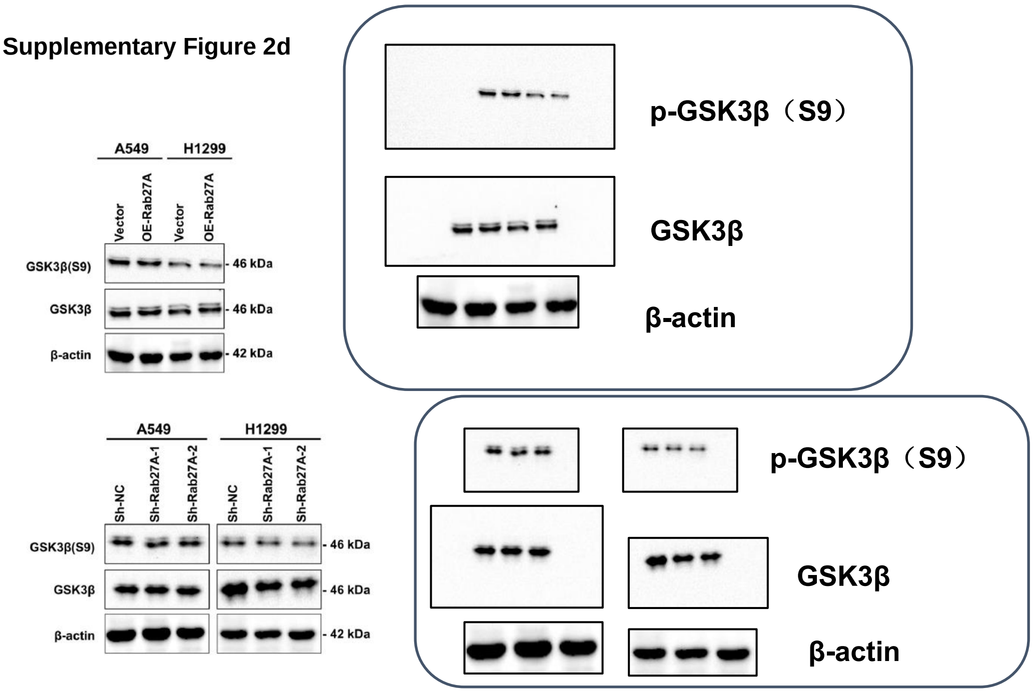

Supplementary Figure 3b

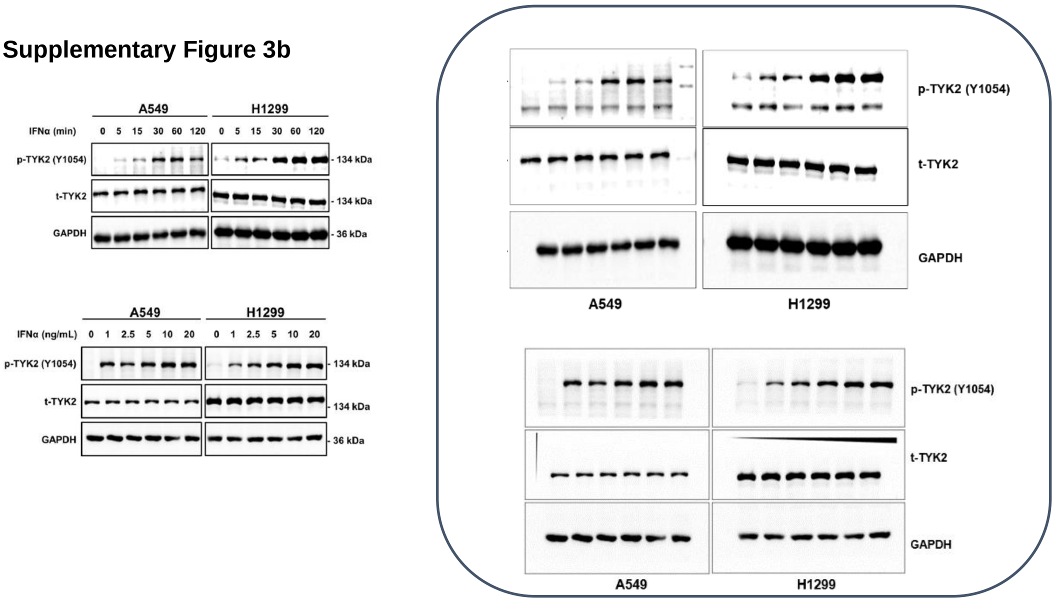

Supplementary Figure 6e

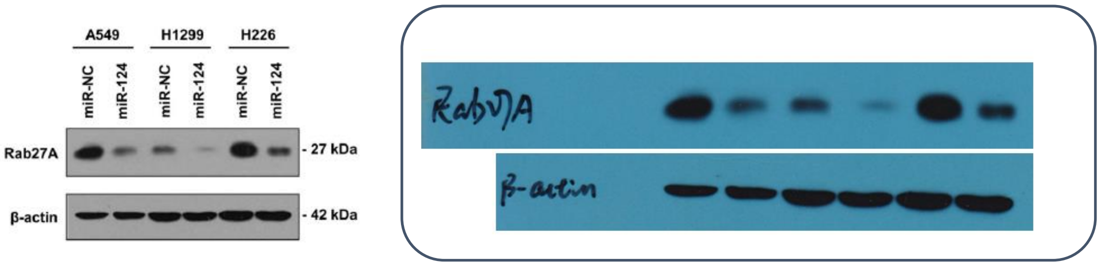

Supplementary Figure 8 The gating strategy of flow cytometry analysis for cell cycle in Supplementary Figure1c and Supplementary Figure 4b was provided.

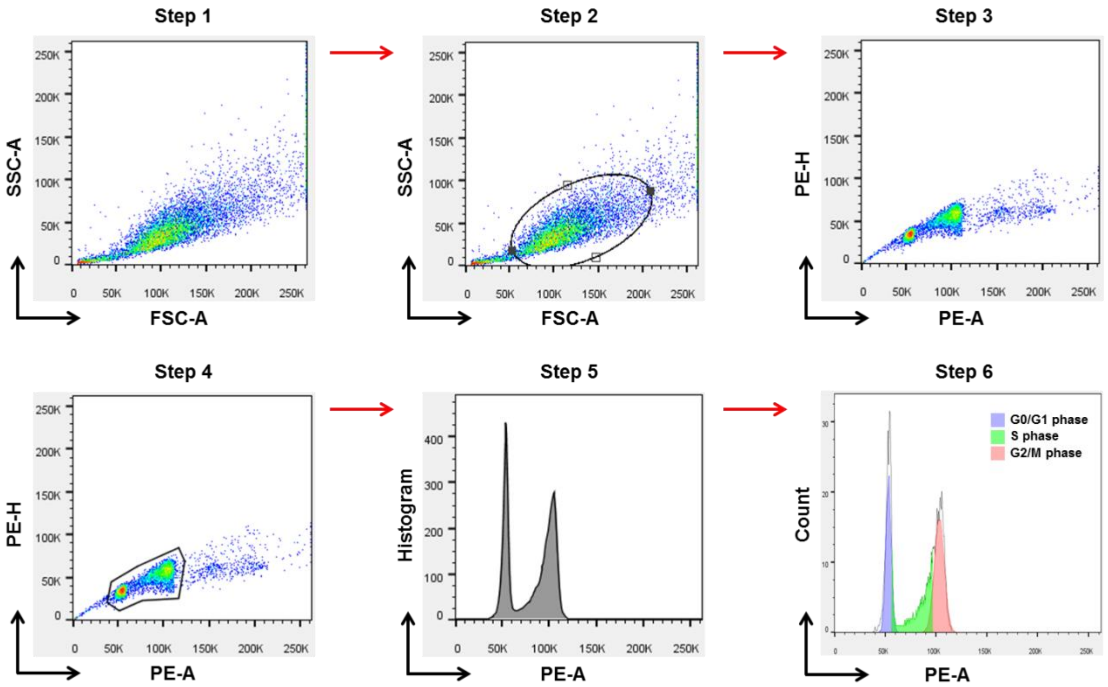

Supplementary Tables

Supplementary Table 1. Differentially expressed proteins between Rab27A-knockdown and control cells based on protein mass spectrometry

Accession	Symbol	Coverage [%]	Peptides	# PSMs	# Unique Peptides	# AAs	Score Sequest HT
P51159	RAB27A	77	25	86	25	221	312.18
Q9BVA1	TUBB2B	61	30	54	1	445	214.65
P04350	TUBB4A	66	28	51	3	444	200.33
Q96C24	SYTL4	29	18	22	18	671	74.82
Q92616	GCN1	11	18	19	18	2671	58.44
Q9BUF5	TUBB6	23	10	13	4	446	46.75
Q9UJS0	SLC25A13	18	8	11	8	675	38.78
Q53GQ0	HSD17B12	22	5	6	5	312	21.05
P53007	SLC25A1	21	6	7	6	311	20.72
O14828	SCAMP3	14	4	6	4	347	20.19
Q5VYK3	ECPAS	4	6	7	6	1845	18.15
Q86VP6	CAND1	6	5	6	5	1230	17.46
P46977	STT3A	5	3	5	3	705	15.74
O00148	DDX39A	19	4	4	3	427	15.53
Q9NVH1	DNAJC11	7	3	5	3	559	14.77
Q8TDW5	SYTL5	8	5	5	5	730	13.75
Q5SRD1	TIMM23B	16	3	4	3	257	13.66
Q96CS3	FAF2	14	4	4	4	445	13.54
P51148	RAB5C	19	3	4	3	216	13.51
P61619	SEC61A1	9	4	4	4	476	12.65
O95373	IPO7	3	2	3	2	1038	12.38
P04844	RPN2	3	1	3	1	631	12.07
P49593	PPM1F	11	4	4	4	454	11.8
O75396	SEC22B	30	3	3	3	215	10.94
Q02978	SLC25A11	16	4	4	4	314	10.81
Q9HCU5	PREB	7	2	3	2	417	10.15
Q96A33	CCDC47	7	2	3	2	483	10.08
Q9Y5M8	SRPRB	20	3	3	3	271	10.07
P50402	EMD	24	3	3	3	254	9.31
P02786	TFRC	5	3	3	3	760	9.12
Q969V3	NCLN	8	3	3	3	563	9.04
O75964	ATP5MG	19	2	2	2	103	8.4
Q8TEX9	IPO4	4	3	3	3	1081	8.29
Q01813	PFKP	5	3	3	2	784	8.1
P50454	SERPINH1	10	3	3	3	418	8.08
P11166	SLC2A1	6	2	2	2	492	8.03

P51571	SSR4	12	2	3	2	173	7.8
Q15758	SLC1A5	6	2	2	2	541	7.1
P30519	HMOX2	10	2	2	2	316	7.02
O95433	AHSA1	12	2	2	2	338	6.91
Q9NPL8	TIMMDC1	5	1	2	1	285	6.33
Q96G23	CERS2	6	1	1	1	380	6.24
O75880	SCO1	5	1	2	1	301	5.88
Q9NWW5	CLN6	7	1	1	1	311	5.62
Q9NX63	CHCHD3	13	2	2	2	227	5.36
O75947	ATP5PD	10	1	2	1	161	4.87
P07858	CTSB	5	1	1	1	339	4.8
Q15070	OXA1L	5	2	2	2	435	4.66
Q9UBM7	DHCR7	4	2	2	2	475	4.65
P11310	ACADM	5	2	2	2	421	4.13
Q8N8S7	ENAH	4	1	1	1	591	3.72
O95394	PGM3	2	1	1	1	542	3.62
O60220	TIMM8A	18	1	1	1	97	3.53
P51153	RAB13	7	1	1	1	203	3.1
P43490	NAMPT	4	1	1	1	491	3.09
Q14318	FKBP8	4	1	1	1	412	3.02
Q9BSD7	NTPCR	6	1	1	1	190	2.92
Q9BU23	LMF2	2	1	1	1	707	2.69
O14773	TPP1	2	1	1	1	563	2.55
Q6Y1H2	HACD2	5	1	1	1	254	2.52
Q8WVM8	SCFD1	2	1	1	1	642	2.39
Q14739	LBR	2	1	1	1	615	2.37
Q9Y6G9	DYNC1LI1	3	1	1	1	523	2.32
P51617	IRAK1	2	1	1	1	712	2.25
P38606	ATP6V1A	1	1	1	1	617	2.24
P10155	RO60	2	1	1	1	538	2.14
Q9HA47	UCK1	3	1	1	1	277	2.13
Q9HD45	TM9SF3	2	1	1	1	589	2.11
P38117	ETFB	3	1	1	1	255	2.05
Q9NRP0	OSTC	8	1	1	1	149	2.05
O95573	ACSL3	3	1	1	1	720	2.02
P00387	CYB5R3	6	1	1	1	301	2.01

Supplementary Table 2. Plot map of Human Phosphorylation Multi-Pathway Profiling Array

ARRAY 1 (MAPK Pathway)

		A	B	C	D	E	F	G	H
Each antibody is spotted in duplicate vertically	1<br>2	POS	POS	NEG	NEG	IC	CREB (S133)	ERK1 (T202/Y204) ERK2 (Y185/Y187)	IC
	3<br>4	IC	HSP27 (S82)	JNK (T183)	MEK (S217/221)	MKK3 (S189)	MMK6 (S207)	MSK2 (S360	IC
	5<br>6	p38 (T180/Y182)	P53 (S15)	IC	RSK1 (S380)	RSK2 (S386)	NEG	NEG	POS

ARRAY 2 (AKT Pathway)

		A	B	C	D	E	F	G	H
Each antibody is spotted in duplicate vertically	1<br>2	POS	POS	NEG	NEG	AKT (S473)	AMPKa (T172)	BAD (S112)	4E-BP1 (T36)
	3<br>4	IC	GSK3a (S21)	GSK3b (S9)	mTOR (S2448)	p27 (T198)	IC	P70S6K (T421/S424)	PDK1 (S241)
	5<br>6	PRAS40 (T246)	PTEN (S380)	RAF-1 (S301)	RPS6 (S235/236)	IC	IC	NEG	POS

ARRAY 3 (JAK/STAT Pathway)

		A	B	C	D	E	F	G	H
Each antibody is spotted in duplicate vertically	1<br>2	POS	POS	NEG	EGFR (Ser1070)	JAK1 (Tyr1022)	JAK2 (Tyr1007/1008)	SHP1 (Ser591)	SHP2 (Tyr542)
	3<br>4	Src (Tyr419)	Stat1 (Ser727)	Stat2 (Tyr689)	Stat3 (Tyr705)	Stat5 (Tyr694)	Stat6 (Tyr641)	TYK2 (Tyr1054)	POS

ARRAY 4 (NFkB Pathway)

		A	B	C	D	E	F	G	H
Each antibody is spotted in duplicate vertically	1<br>2	POS	POS	NEG	NEG	ATM (S1981)	eIF2a (S51)	HDAC2 (S394)	HDAC4 (S632)
	3<br>4	IkBa (S32)	MSK1 (S376)	NFkB (S536)	IC	TAK1 (S412)	TBK1 (S172)	ZAP70 (Y292)	POS

ARRAY 5 (TGFb Pathway)

		A	B	C	D	E	F	G	H
Each antibody is spotted in duplicate vertically	1<br>2	POS	POS	NEG	NEG	ATF2 (T69/71)	C-Fos (T232)	c-Jun (S73)	SMAD1 (S463/465)
	3<br>4	SMAD2(S245/250/255)	SMAD4 (T277)	SMAD5 (S463/465)	IC	NEG	NEG	NEG	POS

Supplementary Table 3. Primers used for Rab27A plasmids construction

Primers sequence	5'-3'
Rab27A-CDS-F	AGATGCTAGCATGTCTGATGGAGATTATG
Rab27A-CDS-R	AGATGGATCCTCAACAGCCACATGCCCCT
Sh- Rab27A-1-F	GATCCGCTACAGATGCATGCATATTTCTCGAGAAATATG CATGCATCTGTAGCTTTTTT
Sh- Rab27A-1-R	AATTAAAAAAGCTACAGATGCATGCATATTTCTCGAGAA ATATGCATGCATCTGTAGCG
Sh- Rab27A-2-F	GATCCGAAGGAGTGGTGCGATCAAATCTCGAGATTTGA TCGCACCACTCCTTCTTTTTT
Sh- Rab27A-2-R	AATTAAAAAAGAAGGAGTGGTGCGATCAAATCTCGAGA TTTGATCGCACCACTCCTTCG

CDS, coding sequence; F, forward; R, reverse.

Supplementary Table 4. Targeted SiRNA sequence for RNA interference

siRNA sequence	5'-3'
Si-NC-sence	UUCUCCGAACGUGUCACGUTT
Si-NC-antisence	ACGUGACACGUUCGGAGAATT
Si-HSPA5-1-sence	GGUGGGCAAACAAAGACAUTT
Si-HSPA5-1-antisence	AUGUCUUUGUUUGCCCACCTT
Si-HSPA5-2-sence	GGUACUGCUUGAUGUAUGUTT
Si-HSPA5-2-antisence	ACAUACAUCAAGCAGUACCTT
Si-STAT1-1-sence	GCUGGAUGAUCAAUAUAGUTT
Si- STAT1-1-antisence	ACUAUAUUGAUCAUCCAGCTT
Si-STAT1-2-sence	GCCCAAUGCUUGCUUGGAUTT
Si- STAT1-2-antisence	AUCCAAGCAAGCAUUGGGCTT
Si-STAT2-1-sence	CAGGAAUCCUCCUCAAUUATT
Si- STAT2-1-antisence	UAAUUGAGGAGGAUUCCUGTT
Si-STAT2-2-sence	GGCCGAUUAACUACCCUAATT
Si- STAT2-2-antisence	UUAGGGUAGUUAAUCGGCCTT

SiRNA: Short interfering RNA

Supplementary Table 5. Primers used for qRT-PCR

Primers sequence	5'-3'
Rab27A-qRT-F	ACAACAGTGGGCATTGATTTCA
Rab27A-qRT-R	AAGCTACGAAACCTCTCCTGC
HSPA5-qRT-F	CATCACGCCGTCCTATGTCG
HSPA5-qRT-R	CGTCAAAGACCGTGTTCTCG
MMP7-qRT-F	GAGTGAGCTACAGTGGGAACA
MMP7-qRT-R	CTATGACGCGGGAGTTTAACAT
MMP9-qRT-F	TGTACCGCTATGGTTACACTCG
MMP9-qRT-R	GGCAGGGACAGTTGCTTCT
E-cadherin-qRT-F	CGAGAGCTACACGTTCACGG
E-cadherin-qRT-R	GGGTGTCGAGGGAAAAATAGG
N-cadherin-qRT-F	TCAGGCGTCTGTAGAGGCTT
N-cadherin-qRT-R	ATGCACATCCTTCGATAAGACTG
STAT1-qRT-F	CAGCTTGACTCAAAATTCCTGGA
STAT1-qRT-R	TGAAGATTACGCTTGCTTTTCCT
STAT2-qRT-F	CCAGCTTTACTCGCACAGC
STAT2-qRT-R	AGCCTTGGAATCATCACTCCC
β-actin-qRT-F	CACAGAGCCTCGCCTTTGCC
β-actin-qRT-F	CATGCCGGAGCCGTTGTCG

qRT-PCR: Quantitative Real-time PCR

Supplementary Table 6. List of antibodies with catalog numbers and dilution

Antibody	Catalog number	Dilution
Rab27A Rabbit Monoclonal antibody	69295S (Cell Signaling Technology)	1:1000
Rab27A Mouse Monoclonal antibody	ab55667 (Abcam)	1:2000
β-actin Mouse Monoclonal antibody	66009-1-Ig (Proteintech)	1:100000
PCNA Mouse Monoclonal antibody	60097-1-Ig (Proteintech)	1:5000
CD9 Rabbit Monoclonal antibody	13403S (Cell Signaling Technology)	1:1000
CD81 Mouse Monoclonal antibody	sc-166029 (Santa Cruz	1:1000
TSG101 Mouse Monoclonal antibody	67381-1-Ig (Proteintech)	1:5000
GM130 Rabbit Polyclonal antibody	11308-1-AP (Proteintech)	1:5000
Calnexin Rabbit Polyclonal antibody	10427-2-AP (Proteintech)	1:5000
TYK2 Rabbit Monoclonal antibody	14193S (Cell Signaling Technology)	1:1000
p-TYK2 (Tyr1054/1055) Rabbit Monoclonal	68790S (Cell Signaling Technology)	1:1000
HSPA5 Mouse Monoclonal antibody	66574-1-Ig (Proteintech)	1:5000
MMP2 Rabbit Polyclonal antibody	10373-2-AP (Proteintech)	1:1000
MMP9 Rabbit Monoclonal antibody	13667S (Cell Signaling Technology)	1:1000
N-cadherin Rabbit Polyclonal antibody	22018-1-AP (Proteintech)	1:5000
Snail Mouse Monoclonal antibody	3895S (Cell Signaling Technology)	1:1000
Cyclin D1 Rabbit Monoclonal antibody	55506S (Cell Signaling Technology)	1:1000
Cyclin A2 Rabbit Polyclonal antibody	18202-1-AP (Proteintech)	1:5000
GSK3β Rabbit Monoclonal antibody	12456T (Cell Signaling Technology)	1:1000
p-GSK-3β(Ser9) Rabbit Monoclonal antibody	5558T (Cell Signaling Technology)	1:1000
